# Degumming and characterization of *Bombyx mori* and non-mulberry silks from Saturniidae silkworms

**DOI:** 10.1038/s41598-023-46474-5

**Published:** 2023-11-09

**Authors:** Theresa Schmidt, Nils Puchalla, Marcel Schendzielorz, Annemarie E. Kramell

**Affiliations:** Department of Organic Chemistry, Martin-Luther-University Halle-Wittenberg, Kurt-Mothes-Straße 2, 06120 Halle, Germany

**Keywords:** Biomaterials, Zoology

## Abstract

In this study, cocoons and degummed silk samples of *Bombyx mori* and twenty Saturniidae species of the genera *Actias*, *Attacus*, *Argema*, *Antheraea*, *Caligula*, *Callosamia*, *Cricula*, *Epiphora*, *Hyalophora*, *Loepa*, *Samia* and *Saturnia* are studied to gain an insight into their morphology, chemical composition and physical structure. For this purpose, silk samples are characterized by optical microscopy and FTIR spectroscopy in attenuated total reflection mode (ATR-FTIR spectroscopy). Furthermore, degummed silk samples are analyzed for their amino acid (AA) composition by GC-FID. In the course of method development, various degumming methods are tested using alkalis, citric acid, enzymes and detergents. A mixture of 0.1% sodium carbonate and 2.5% ethylenediamine proves to be an effective agent for degumming Saturniidae and *B. mori* cocoons. After hydrolysis of the fibroin filaments with 6 N hydrochloric acid and derivatization with propyl chloroformate, fifteen AAs are identified and qualified. This method shows a satisfactory overall analytical performance with an average recovery rate of 95% at the medium concentration level. The chemical composition of the different silks was considered comparatively. Within a genus, the analyses usually show a high degree of similarity in AA composition and the resulting structural indices, whereas differences are found between genera.

## Introduction

Silk is one of the most exclusive fibers in the world and has been used for textile production for several thousand years. The earliest direct biomolecular evidence for the existence of silk is from tombs dated 9000–8500 BP in Jiahu, China^1^. For centuries, the domesticated silkworm [*Bombyx mori* L. (BM), family Bombycidae], also known as mulberry silk moth, has been the most important producer of silk. These mulberry feeding silkworms originated from the Chinese wild silkworm *Bombyx mandarina* Moore that occurs throughout Asia, where modern sericulture and silkworm domestication were initiated^2,3^. Beside BM and *B. mandarina*, several wild or semi-domesticated moth larvaes produce silks with remarkable mechanical properties that have attracted large interest in the scientific world^4–7^. These non-mulberry feeding silkworm species are diverse and have a wide distribution throughout the world. Among non-mulberry silk moth the most well-known species, belonging to the family Saturniidae, are *Antheraea pernyi* Guérin-Méneville [APe, Chinese temperate (oak) tasar/tussah], *A. proylei* Jolly (Indian oak tasar, hybrid of *A. roylei* Moore and APe^8^), *A. mylitta* Drury (AM, Indian tropical tasar), *A. assamensis* Helfer (Indian muga), *Samia cynthia* Drury (SCy, Indian eri), and *A. yamamai* Guérin-Méneville (Japanese oak silk). Some species have great economic importance and are cultivated not only for textile production. For instance, APe, which uses oaks (various *Quercus* species) as host plants, is also used in traditional medicine, cosmetic products as animal feed, farm fertilizer and a food source for human consumption^9–11^. To meet this demand, thousands of hectares of oak are under cultivation today in China, India and Korea for silk, egg, larva and pupae production^10^. In contrast, silks from wild silk moth such as *Pachypasa otus* Drury (Lasiocampidae family), the source of Coan silk, have only historical significance^12^.

A cocoon, whose primary function is to protect the developing moth against its natural enemies and climate conditions, is a multilayer composite material composed of two proteins, namely fibroin and sericin. Sericin, also known as gum, is a glue-like protein, which coats and binds together filaments of the protein fibroin into an intact cocoon. The structure and composition of the water-soluble sericin coating of BM has been the subject of several studies^13–15^. The sericin coating composed of two or more layers that cover the outside of the fibroin filaments. These layers of sericin consists mainly of AAs with a polar side chain such as Ser, Asp and Thr, with the amount of polar AAs in the sericin layers gradually decreasing from the outer to the inner layer. In production of silk threads and fabrics, sericin must be removed to obtain glossy, soft, smooth and dyeable materials. Conventional degumming processes, during which sericin is fully/partially hydrolyzed or solubilized, are based on the use of soap, alkalis, organic acids, enzymes or detergents^16,17^. In addition, various extraction processes, such as using water at high temperature and pressure can be used for the removal of sericin^18^. In contrast to sericin, fibroin is insoluble in water, has a fibrous nature and contains a high proportion of amino acids (AAs) with non-polar side chains^19^. Silk fibroin from BM is composed of three proteins, heavy chain fibroin (FibH, 350 kDa), light chain fibroin (FibL 26 kDa) and P25 (also known as fibrohexamerin, 30 kDa), and consists mainly of Ala- (alanine) and Gly- (glycine) rich repetitive motifs^19^. The BM fibroin elementary unit consists of six disulfide-linked FibH and FibL dimers and one P25 glycoprotein molecule^20^. However, the AA sequence and composition of silk fibroin from non-mulberry silkworms differs from BM^21,22^. In this context, the chemical composition of various *Antheraea* species was studied in particular^23^. For instance, fibroin of commercial *Antheraea* species such as APe*,* AM and *A. assamensis* is characterized by a significantly higher content of Ala as well as a higher ratio of basic to acidic and hydrophilic to hydrophobic AAs compared to BM fibroin^7,24,25^. In addition, significant differences in mechanical properties and structural morphologies were found between silks of BM and other species^5,21^. Silk filaments of BM, for example, have a triangular cross-sectional shape, while wild silk is mostly elongated rectangular or a wedge-shaped^5,24^. In order to better understand the structural and chemical composition of non-mulberry silks, different techniques are used, e.g. (Wide-angle) X-ray diffraction, IR and Raman spectroscopy^21,23,24,26–28^. In addition, AA analyses using an Amino Acid Analyzer (AAA) as well as MS analyses of trypsinized cocoons or silks are used to characterize and identify silk proteins^13,22,23,28^.

In this study, we report on the development and validation of a GC-FID method for the identification and quantification of fifteen AAs in silk fibroin of twenty-one silkworm species. Cocoons of BM and non-mulberry silkworms in the tribes *Saturniini* and *Attacini* (family Saturniidae) were degummed and the obtained fibroin filaments were analyzed for their AA composition. In this context, different degumming methods were tested, using optical microscopy and FTIR spectroscopy in attenuated total reflection mode (ATR-FTIR spectroscopy) as well as degumming ratio to monitor the degumming process and characterize the degummed silks. AA compositions, morphologies and ATR-FTIR spectra of the silk samples were compared and differences between silks produced by Saturniidae or Bombycidae moths were discussed. The aim of this study is to contribute basic knowledge on degumming of BM and Saturniidae cocoons, the chemical composition and the physical structure of the degummed silk filaments. Furthermore, the potential of AA analyses, microscopic and ATR-FTIR spectroscopic studies to distinguish between Saturniidae silks was investigated.

## Experimental section

### Chemicals and material

L-Alanine (Ala; 99%), L-isoleucine (Ile; 99%), L-proline (Pro; 99%), L-cysteine (Cys; > 98%), L-lysine (Lys; 98%), L-norleucine (Nle; 99%) and 3-picoline (99%) were obtained from Alfa Aesar. Glycine (Gly; ≥ 99%) were purchased from Tokyo Chemical Industry (TCI); L-leucin (Leu; ≥ 99%), L-valine (Val; ≥ 99%), L-glutamic acid (Glu; ≥ 99%), L-aspartic acid (Asp; ≥ 99%), L-threonine (Thr; ≥ 99%), L-phenylalanine (Phe; ≥ 99%) and L-methionine (Met; ≥ 99%) from CARL ROTH GmbH + Co. KG; L-serine (Ser; > 99%), L-histidine (His; ≥ 99%) sodium carbonate (Na_2_CO_3_; anhydrous), sodium hydrogen carbonate (NaHCO_3_), citric acid (99%), ethylene diamine (≥ 99%), phenol (≥ 99%), 3,3′-dithiodipropionic acid (DTDPA, 99%) and sericin BM (silkworm) from Sigma-Aldrich; tryptophane (Trp; 99%), propyl chloroformate (98%) and *n*-Propanol (> 99%, extra pure) from Acros Organics; isooctane (ACS, Reag. Ph. Eur) from VWR Chemicals; chloroform (≥ 99.8%), concentrated hydrochloric acid (HCl; 37%) and sulphuric acid (≥ 95%) from Fisher Scientific and papain from carica papaya (3.0 U mg ^−1^) was bought from Fluka Chemicals. Handcrafted Savon de Marseille soap was obtained from the Savonnerie Fer à Cheval; Marseille, France. The industrial degumming detergent Perlavin LMO in combination with the sequestering solution Periquest APG (alkyl polyglycoside) was bought from Dr. Petry—Textile Auxiliaries, Reutlingen, Germany. The pineapples were purchased from a local market.

### Cocoons and silk samples

BM cocoons were obtained from World of Butterflies & Moths (Lincolnshire, England), a Chinese web store and from the Francke Foundations in Halle/Saale (silk worms were cultivated within the framework of a pedagogical project). Degummed BM silk and cocoons of AM (Indian tasar silkmoth) were purchased from Seidentraum (organic peace silk, Sternenfels, Germany). Cocoons of *Attacus atlas* L. (AtA) were obtained from the butterfly house of Jonsdorf (Germany); other non-mulberry silkworm cocoons from World of Butterflies & Moths (Lincolnshire, England) or from Worldwide Butterflies (England).

### Optical microscopy (OM) and ATR-FTIR spectroscopy

The surface morphology of the cocoons (outside and inside) and degummed silken filaments was characterized using a VHX-6000 digital microscope from KEYENCE. For ATR-FTIR analysis, a Perkin Elmer UATR two spectrometer was used. ATR-FTIR spectra were acquired with 64 or 256 scans over the range of 4000 to 400 cm^−1^ at a spectral resolution of 4 cm^−1^. All samples were scanned on 2 to 5 different positions. Background was collected each time before all ATR-FTIR spectra of silk samples were collected. Spectral data analysis, including baseline correction, smoothing and deconvolution of amide I bands (1600–1700 cm^−1^) were performed using Spectrum 10 software (Perkin Elmer) and PeakFit 4.12 (Systat Software Inc.) according to the literature^29–31^. The numbers and positions of peaks were defined from the results of second derivatives spectra and fixed during the deconvolution process. The data (e.g. β-sheet content) obtained from the spectra were the mean and standard deviations taken from separate deconvolutions from at least three separate samples. Assignment of adsorption peaks in the amide I band: the peak from 1619 to 1624 cm^−1^ is assigned to β-sheet conformation; the small peak from 1687 to 1692 cm^−1^ to β-turn conformation of the hairpin-folded antiparallel β-sheet structure; and the peak centered at 1653–1662 cm^−1^ to random coil, helical conformation, or both.

### Degumming

Sericin removal was performed by different methods: alkaline or acidic methods with Na_2_CO_3_, NaHCO_3_, ethylenediamine or citric acid, enzymatic processes with papain or pineapple juice and methods with detergents such as Marseille soap or industrially used Perlavin LMO (modified according to^16,17,28,32,33^. After degumming, the fibers were immediately rinsed thoroughly with distilled water until a neutral pH value was achieved and dried at 100 °C until constant weight was reached. The degumming ratio D_r_ (%), which correspond to the amount of sericin and non-sericin components (including wax, pigments, sugars and other impurities) removed by different degumming treatment, was expressed in terms of percentage weight loss using the weights of the dried silk samples before (W_0_) and after (W_1_) degumming (see Feng et al.^34^). For the D_r_ calculation, the following Eq. (1) was used:

1 $$D_{r} \left( \% \right) = \frac{{W_{0} - W_{1} }}{{W_{0} }}*100$$

In addition, microscopic and ATR-FTIR spectroscopic examinations were carried out to assess the efficiency of the degumming process. Degumming methods were first applied to cocoons of BM, and subsequently the studies were extended to non-mulberry silkworm cocoons, e.g. of AtA and AM. Pupae, plant parts and coarse impurities were removed with tweezers before degumming. All experiments were repeated three times.

In alkaline method, cocoons were treated with an aqueous solution of Na_2_CO_3_ (1 g L^−1^) at 95 °C for 30–90 min. To optimize the degumming process, the reaction time (30, 60, 90, 120 min), concentration of the Na_2_CO_3_ solution (between 0.1 and 5 g L^−1^) and the temperature of the water bath (80 °C, 90 °C and 95 °C) were varied.

Furthermore, cocoons were degummed at 95 °C for 30–120 min using different concentrations of ethylenediamine (2.5 and 10% v/v) or NaHCO_3_ (0.5 and 1% w/v). As alternative method, cocoons were boiled at 95 °C in an aqueous solution containing Na_2_CO_3_ (1 g L^−1^) and ethylenediamine (2.5% w/w). This study was conducted for 30, 60, 90 and 120 min. The recommended reaction time for BM cocoons is 30–60 min. However, reaction times of up to 300 min are required for decoating of non-mulberry cocoons.

In addition to alkaline degumming described above, an acidic approach using citric acid (1 and 2 g L^−1−^; reaction time: 120, 240 min; temperature of the degumming bath: 95 °C), an enzyme-based method with papain (1 g L^−1^; reaction time: 120, 240 min; temperature of the degumming bath: 90 °C; pH value of the degumming bath: 6) and the use of an industrial detergent (1 g L^−1^ Periquest APG as a nonionic surfactant and 5 g L^−1^ Perlavin LMO as a detergent based on natural soaps with additional fiber-protective components—specifically designed for degumming silk; reaction time: 60, 90 and 120 min; temperature of the degumming bath: 95 °C) were evaluated. Prior to silk degumming with Periquest APG and Perlavin LMO, the degumming bath was adjusted to pH 10 with a NaOH solution.

Conventional degumming methods using Marseille soap (1 and 2 g L^−1^; pH value of the degumming bath: 8–10) or fresh pineapple juice, which is known to be a reservoir of proteolytic enzymes, were also tested. For the conventional soap process, the degumming bath was heated to 95 °C and process was continued for 120 min at this temperature. The degumming using the juice of a pineapple was carried out at 90 °C for 120 and 240 min, respectively. For this purpose, pineapples were peeled, cut and blended using a mixer. The filtrate was used to remove the sericin coating.

### AA analysis by GC-FID

#### Preparation of standard solutions

Stock solutions (50 mM) of (L-)AAs were individually prepared in aqueous hydrochloric acid (0.1 M) and diluted with aqueous hydrochloric acid (0.1 M) to obtain working solutions down to a concentration of 1 mM. Nle (50 mM) was used as an internal standard.

#### Hydrolysis of Silk Fibroin (modified according to Vilaplana et al.^35^)

Hydrolysis of the degummed silk samples (1–10 mg) was performed with hydrochloric acid (1000 µL, 6 N) at 110 °C for 24 h. As stabilization reagents, phenol in water (50 µL, 1%) and 3,3′-dithiodipropionic acid (50 µL, 1%) in an aqueous solution of NaOH (0.2 M) were added to all samples. The hydrolysate was concentrated to dryness at 90 °C using a nitrogen stream and the residue was dissolved in hydrochloric acid (200 µL, 0.1 M). Aliquotes (25–200 µL) were used for AA derivatization.

#### AA derivatization with propyl chloroformate (modified according to the EZ:faast kit derivatization protocol from Phenomenex)

Aliquotes of AA working solutions or hydrolysates (25–200 µL) were diluted up to a volume of 225 µL with distilled water. Subsequently, derivatization reagent 1 (100 µL, 77% *n*-propanol and 23% 3-picoline)^36^ was added and the solution was thorough vortexed for 20 s. In the next step, derivatization reagent 2 (70 µL, 17.4% propyl chloroformate, 11% isooctane and 71.6% chloroform)^36^ was added, the solution was mixed again for 20 s and remained at room temperature for 1 min. Then the solution was heated to 40 °C for 3 min and vortexed rigorous for 20 s. Finally, isooctane (250 µL) was added and it was mixed again. An aliquote of the organic phase (2.5 µL) was injected into the gas chromatograph.

#### GC-FID analysis

Samples were analyzed with a Shimadzu gas chromatograph GC-2025, equipped with an AOC-20i injector (injection volume: 2.5 µL, split mode, split ratio 15:1), an AOC-20 s autosampler and a flame ionization detector (FID; hydrogen and air flows were set at 40 and 400 mL min^−1^). Chromatographic separation was performed on a Zebron ZB-50 column (15 m × 0.25 mm × 0.15 µm, Phenomenex). The injection port was held at 320 °C and the detector operated at 310 °C. The column oven temperature was initially set to 100 °C, held for 30 s and then ramped with 20 °C min^−1^ to 300 °C, held for 6 min; the carrier gas was nitrogen at a constant column flow of 1.10 mL min^−1^ (linear velocity: 35.7 cm s^−1^). All analyses were performed in triplicate.

#### Principal component analysis (PCA)

PCA of AA composition was performed with Gly and Ala content as well as the amount of polar and non-polar AA residues as independent variables, using Origin 2019 (OriginLab Corporation, US). The first two components account for 98% of the variance of the data.

### Validation experiments

#### Specificity

To determine the specificity of the method, a mixture of all AAs studied was measured by GC-FID after derivatization with PCF [for retention times see Table S1, Supplementary Information (SI)]. Furthermore, hydrolysates of degummed BM and non-mulberry silkworm silk as well as blank samples (without AA or protein addition) were analyzed by GC-FID.

#### Linearity, limit of detection (LOD) and limit of quantification (LOQ)

For the calibration, solutions were prepared with different mixtures of proteinogenic AAs and Nle [AAs: in the range of min 0.6 to max 20.4 mM with Nle as internal standard (50 µL, 50 mM); additionally extended concentration range for Ala and Gly: 6.6–24.8 mM with Nle as internal standard (110 µL, 50 mM)]. Each calibration solution was measured three times and analyses were executed with average peak area ratios of the respective AA and Nle. The linearity of each calibration function was tested with the Mandel’s test. Limit of detection (LOD) and limit of quantification (LOQ) were determined by means of a calibration curve method according to DIN 32645^37^. These validation data are shown in Table S1, SI.

#### Precision

For determining the repeatability, six replicate measurements were carried out for two different concentrations (Table S1, SI). For this purpose, solutions containing respective AAs and Nle as internal standard were prepared, derivatized and analyzed with GC-FID. Ala and Gly were analyzed separately from the other AAs due to the high concentration differences. For the interpretation of the repeatability, the relative standard deviation (RSD) of the respective AA concentration was used. For determining the method precision, six BM silk samples (10 mg), degummed with method 4–2 (see Table 1), were hydrolyzed and independently derivatized in the presence of the internal standard Nle. Aliquots (2.5 µL) of the organic phases were analyzed by GC-FID. For the interpretation of the method precision, the RSD of the AA concentrations was used (Table S1, SI). Furthermore, Dixon’s Q test and Neumann trend test were applied for the identification of outliers or trends.

**Table 1:** Degumming ratio of different degumming methods using mulberry and non-mulberry silkworm cocoons.

Degumming method	Method ID	D_r_ [%]		
		BM	AM	AtA
Alkaline				
Na_2_CO_3_ (1 g L^−1^), 30 min, 95 °C	1–1	34	–	
Na_2_CO_3_ (1 g L^−1^), 60 min, 95 °C	1–2	31	–	
Na_2_CO_3_ (1 g L^−1^), 90 min, 95 °C	1–3	28	–	–
NaHCO_3_ (0.5%), 120 min, 95 °C	2–1	28	–	
NaHCO_3_ (1%), 120 min, 95 °C	2–2	29	–	
Ethylenediamine (2.5%), 30 min, 95 °C	3–1	36	–	
Ethylenediamine (2.5%), 60 min, 95 °C	3–2	29	23	19
Ethylenediamine (2.5%), 90 min, 95 °C	3–3	30	23	19
Ethylenediamine (2.5%), 120 min, 95 °C	3–4	34	–	–
Ethylenediamine (10%), 120 min, 95 °C	3–5	38	–	–
Na_2_CO_3_ (1 g L^−1^) and ethylenediamine (2.5%), 30 min, 95 °C	4–1	32	28	–
Na_2_CO_3_ (1 g L^−1^) and ethylenediamine (2.5%), 60 min, 95 °C	4–2	31	24	16
Na_2_CO_3_ (1 g L^−1^) and ethylenediamine (2.5%), 90 min, 95 °C	4–3	31	24	17
Na_2_CO_3_ (1 g L^−1^) and ethylenediamine (2.5%), 120 min, 95 °C	4–4	–	–	22
Acidic				
Citric acid (1 g L^−1^), 120 min, 95 °C	5–1	9	–	–
Citric acid (1 g L^−1^), 240 min, 95 °C	5–2	11	–	–
Citric acid (2 g L^−1^), 120 min, 95 °C	5–3	11	–	–
Detergent-based				
Marseille soap (1 g L^−1^), 120 min, 95 °C	6–1	23	–	–
Marseille soap (2 g L^−1^), 120 min, 95 °C	6–2	28	–	–
Periquest APG (1 g L^−1^) and Perlavin LMO (5 g L^−1^), 60 min, 95 °C	7–1	34	11	16
Periquest APG (1 g L^−1^) and Perlavin LMO (5 g L^−1^), 90 min, 95 °C	7–2	34	10	18
Periquest APG (1 g L^−1^) and Perlavin LMO (5 g L^−1^), 120 min, 95 °C	7–3	32	–	–
Enzyme-based				
Papain (1 g L^−1^), 120 min, 90 °C	8–1	23	–	–
Papain (1 g L^−1^), 240 min, 90 °C	8–2	25	–	–
Pineapple juice, 120 min, 90 °C	9–1	9	–	–
Pineapple juice, 240 min, 90 °C	9–2	18	–	–

#### Recovery

The calculation of the recovery rate for each AA was performed for three concentration levels with a number of three replicates (Table S1, SI). For this purpose, degummed silken filaments of BM (10 mg, degumming method 4–2, see Table 1) were spiked with a defined AA concentration, hydrolyzed with hydrochloric acid at 110 °C for 24 h and derivatized with PCF/*n*-propanol as described above. Hydrolysis, derivatization and GC-FID analysis of the spiked silk samples were performed independently of each other on different days. The calculated AA concentrations were compared with the target concentrations and the recovery rate was determined. The average AA concentrations of degummed silken filaments of BM, determined in the course of the method precision, were used for the calculation of the target concentrations.

#### Stability of the standards

An aliquote of a working solution containing Nle as internal standard and AAs studied (equimolar mixture) was derivatized with PCF and stored at room temperature in the autosampler of the gas chromatograph. The solution was examined over a 24 h period and analyses were repeated three times with eight independently prepared solutions. Dixon’s Q test and Neumann trend test were applied for the identification of outliers or trends. The investigation of the long-term stability of AA mixtures in 0.1 M hydrochloric acid, stored at 4 °C in the dark and examined over a 7-week period, was carried out during parallel ongoing studies (see Puchalla^38^).

## Results and discussion

The subjects of the studies were, in addition to BM silks (family Bombycidae), cocoons and degummed fibers from twenty non-mulberry feeding silkworms species of the genera *Actias*, *Attacus*, *Argema*, *Antheraea*, *Caligula*, *Callosamia*, *Cricula*, *Epiphora*, *Hyalophora*, *Loepa*, *Samia* and *Saturnia* which belong to the family Saturniidae. In some cases, several silk samples of the same species, but from different regions were examined.

### Degumming of mulberry and non-mulberry silkworm cocoons

#### Degumming of BM cocoons

Different approaches were compared to remove the globular glue protein sericin from silk filaments of BM (Table 1), using microscopic and ATR-FTIR spectroscopic studies as well as the average degumming ratio D_r_ to evaluate the degumming efficiency. As shown in Table 1, the degumming ratio of BM cocoons using 0.5–1% NaHCO_3_, 0.1% Na_2_CO_3_ and/or 2.5% ethylenediamine as degumming agent for 30–90 min at 95 °C is about 30–35%. A degumming ratio of approximately 30–35% can also be achieved with Marseille soap (2 g L^−1^) or the nonionic surfactant Periquest APG in combination with Perlavin LMO, with degumming in both cases taking place under alkaline conditions (pH 8–10). In contrast, a degumming ratio of 9–18% is observed for the acidic and enzyme-based methods using 0.1–0.2% citric acid or pineapple juice as degumming agent. Since the sericin content on the cocoon shell of commercially available BM varieties is usually about 20–30%^14,39^, a degumming ratio < 20% indicates incomplete degumming. Microscopic examination showing residual sericin on the silken filaments degummed with 0.1–0.2% citric acid (D_r_ 9–11%) or pineapple juice (D_r_ 9–18%) confirms incomplete sericin removal (Figure S1A, SI). A degumming rate of 25% is obtained when using papain for 240 min at 90 °C. However, traces of sericin can be observed on some filaments. To increase the degumming efficiency, for example, the use of papain in the presence of urea or treatment with 15–30% citric acid would be conceivable (see Biswal et al.^16^).

In agreement with previous experiments^39^, the determined degumming ratio of the alkaline and detergent-based methods of about 30–35% indicates complete removal of the sericin gum from the silk filaments. Microscopic observations show that silken filaments degummed with 0.1% Na_2_CO_3_ and/or 2.5% ethylenediamine for 30–90 min at 95 °C or using Periquest APG/ Perlavin LMO for 60–120 min at 95 °C are smooth and without residual sericin (Figure S1B, SI). And also during the treatment with Marseille soap (2 g L^−1^) or 0.5–1% NaHCO_3_ for 120 min at 95 °C, twin silk filaments were transformed into monofilament structure. ATR-FTIR spectra of silken filaments treated with these degumming agents show prominent bands at 975, 998 and 1695 cm^−1^ assigned to a − Gly − Ala − peptide backbone and β-sheets indicating a high level of protein crystallinity (Figure S2, SI). Since fibroin has a higher degree of crystallinity than sericin due to stacked β-sheets, the relative intensity of β-sheet peaks serve as indicators of degumming efficiency (see Refs.^16,26^). Studies on untreated BM cocoons show that these bands are masked by the presence of a sericin coating. Furthermore, the intensity of the signature peaks for sericin at around 1400 cm^−1^ and 1070 cm^−1^ is significantly reduced for all degummed samples compared to the untreated cocoons^40^.

In addition, investigations on the AA composition of degummed silk samples were carried out (see AA analysis by GC-FID). For silken filaments degummed with alkaline and detergent-based methods, with the exception of samples treated with Marseille soap, a Ser content of 3.1 to 6.1 mol% was determined (for AA composition see Table S2, SI). The Ser content of silk samples treated with Marseille soap (2 g L^−1^) or papain (240 min) is 7.7 and 6.0 mol%, respectively. In contrast, samples degummed with 0.1–0.2% citric acid or pineapple juice have a Ser content of 9.6–11.1 mol%. A similar trend can be observed for Thr. For instance, alkaline and Periquest APG/ Perlavin LMO-based treatments result in Thr contents of 0.7–1.1 mol%, while Thr contents of 1.5–2.5 mol% were determined for silk samples treated with citric acid or pineapple juice. Also, commercially degummed silk filaments used for textile production have Ser and Thr contents of 5.5 and 0.8 mol%, respectively. Thus, the content of Ser and Thr, both AAs with a polar side chain, is also an excellent indicator of degumming efficiency of BM cocoons. However, it should be noted that treatment of silk samples under extremely acidic and alkaline pH conditions also leads to hydrolysis of peptide bonds in silk fibroin. This hydrolysis occurs mainly in the amorphous spacers within the fibroin chains, which are more sensitive to degradative effects than the crystalline regions. A significant reduction in the AAs Asp, Thr, Ser (polar side chains) Ile and Leu (hydrophobic side chains), which are found mainly in the amorphous regions, is well known for such samples^35^. However, no meaningful decrease in Asx (Asp + Asn, see validation experiments) and Ile content was observed for silk samples degummed by the alkaline and detergent-based methods discussed above (pH value of the degumming baths was in the range of 8 to 10).

Studies by, for example, Dou and Zuo^41^ or Zhao et al. show a correlation between degumming agent concentration, temperature, treatment time and various properties of silk fibers such as mechanical strength, wettability and morphology^39^. Harsh conditions, i.e. long treatment time and/or high concentration of the degumming agent, lead to partial degradation of fibroin fibers and a degumming ratio > 35%, which indicates loss of fibroin protein. For instance, brittle fibers reduced in diameter and a degumming ratio of 38% were observed when BM cocoons were treated with 10% ethylenediamine for 120 min at 95 °C (method 3–5, pH value of the degumming bath: 12, see Table 1).

#### Degumming of non-mulberry silkworm cocoons

In a next step, selected alkaline and Periquest APG/Perlavin LMO-based methods were tested for degumming AM and AtA cocoons (Table 1, Figures S3 and S4, SI), cocoons spun by commercial used silk moth species in the tribes *Saturniini* and *Attacini* (family Saturniidae). AM cocoons have a very hard and compact shell, while AtA cocoons are soft in nature and have an intermediate sericin content compared to BM and AM cocoons^26^. The degumming ratio of AM samples ranged from 28% for filaments degummed with a mixture of Na_2_CO_3_ and ethylenediamine to 10% for cocoons treated with Periquest APG and Perlavin LMO. Degumming ratios of 16–22% were determined for AtA cocoons using ethylenediamine or ethylenediamine in combination with Na_2_CO_3_ for 60–120 min at 95 °C, while values of 16–18% were obtained with Periquest APG/Perlavin LMO-based methods. For AM and AtA samples treated with a mixture of Na_2_CO_3_ and ethylenediamine (AM: 30–90 min, D_r_ 24–28%; AtA: 120 min, D_r_ 22%), the separation of two brins of fibroin is clearly seen under microscope (Figures S3B and S4B, SI). Furthermore, these degummed silk filaments are smooth and not degraded, indicating high degumming efficiency without destruction of the filaments at the macroscopic level. In contrast, detergent-based methods tested result in unseparated brins of fibroin and thus incomplete degumming (AM: D_r_ 10–11%, Figure S3A; AtA: D_r_ 16–18%, Figure S4A, SI). AA analysis shows that Ser content (Ser + Pro, see validation experiments) of AM fibroin ranges from 5.4 to 6.4 mol% and the Thr content ranges from 0.6 to 1.2 mol% for all degumming methods (see Table S3 for AA composition, SI). And also in samples of AtA, Ser (Ser + Pro) and Thr content is not a suitable indicator of degumming efficiency (see Table S4 for AA composition, SI). However, ATR-FTIR spectra of silken filaments of AtA and AM, degummed with ethylenediamine or ethylenediamine/Na_2_CO_3_, show that the intensity of the band at around 1052 cm^−1^ assigned to sericin C–O stretching^26^ is significantly reduced compared to the untreated cocoons and the incompletely degummed samples (Figures S5 and S6, SI).

A mixture of 0.1% Na_2_CO_3_ and 2.5% ethylenediamine was used to degum cocoons of several other non-mulberry feeding silkworm species (tribes *Saturniini* and *Attacini*) that exhibited a wide variety of morphologies and architectures, with porosity ranging from loose meshes to full shells (see Cocoon and fiber morphologies). The progress of the degumming process was monitored by microscopic examinations of the filaments, adjusting the treatment time if necessary. Average degumming ratios, indicative of the removal of adhering constituents (sericin, calcium oxalate, wax, pigemts etc.), and the corresponding treatment times are summarized in Table S5, SI. After degumming, smooth silk filaments were obtained, and twin silk filaments were transformed into monofilament structure. For a comparison between the species, degummed silk samples were characterized by ATR-FTIR spectroscopy and analyzed with respect to their AA composition (see Between-species comparison).

Differences between BM and non-mulberry silkworm cocoons in terms of sericin content, sericin structure and the content of non-protein components such as calcium oxalate crystals are well known and can be attributed to different habitats and host plants^26,42^. In general, harsher conditions are often required for the removal of sericin from silk filaments of non-mulberry silkworms^43^. Thus, ethylenediamine is often used as an effective agent for degumming of non-mulberry silk^25,44^. And also in our studies, ethylenediamine in combination with Na_2_CO_3_ shows a great result as degumming agent for non-mulberry silkworm cocoons.

### Between-species comparison

#### Cocoon and fiber morphologies

As already mentioned, the appearance and dimensions of the examined cocoons are partly very different, whereby also within a genus sometimes substantial differences can be determined. For instance, cocoons spun by different *Antheraea*, *Actias* and *Hyalophora* silk moth species vary significantly in architectural features (Figure S7, SI). In addition, some species such as *Hyalophora cecropia* (HC) are known to produce discrete dimorphic cocoons that are either large and fluffy (baggy) or significantly smaller and tightly woven (compact)^45^. In our study, only baggy HC cocoons were analyzed. Moreover, cocoons of the wild eri silkworm *Samia canningii* (SCa) differ significantly from the cocoons of domestic *Samia* species used commercially for silk production (Figure S7R-T, SI). Non-mulberry silk cocoons mostly have microscale crystals on the outer surface, and some crystals are also seen on the inner surface. The examined Saturniidae silkworm cocoon fibers have a flat and ribbon-like structure with an oval to rectangular cross-section, while the cross section of BM filaments has a triangular shape (Figures S1, S3, S4 and S8, SI).

#### ATR-FTIR analysis

For further characterization, cocoons and degummed silk samples were analyzed by ATR-FTIR spectroscopy (Figures S9-S11, SI). As outlined in previous studies, this approach enables the identification and quantification of, for example, calcium oxalate, serine, polyalanine (A)_n_ β-sheets, polyalanineglycine (AG)_n_ β-sheets, tannins and phenolic compounds present in native silk feedstock and cocoons^26^.

**Figure 1:**
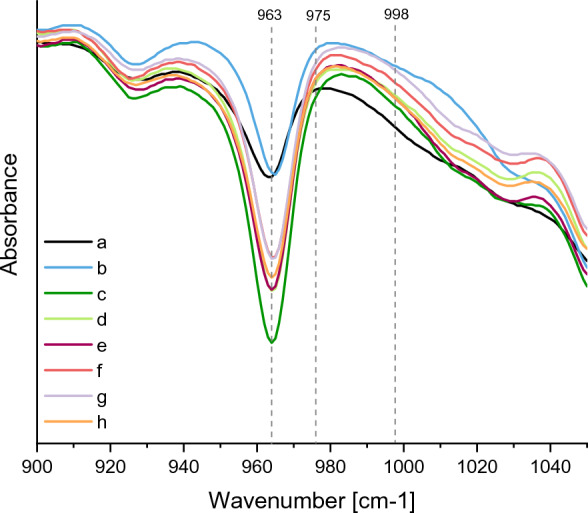
ATR-FTIR spectra of silk degummed with a mixture of 0.1% Na_2_CO_3_ and 2.5% ethylenediamine at 95 °C: (**a**) AcS, (**b**) ArM, (**c**) EB, (**d**) CaP, (**e**) HC, (f) SCy ricini, (**g**) SCa (**h**) Eri silk moth.

In agreement with the studies of Boulet-Audet et al.^26^, an intense band at about 1315 cm^−1^ was observed in the spectra of the untreated *Antheraea* cocoons, especially on the outside of the cocoons, which is assigned to the calcium oxalate vibrational modes and thus indicates a high calcium oxalate content. In contrast, a weak or no characteristic band for calcium oxalate was found on the inner and outer surfaces of the untreated cocoons of *Argema mimosae* (ArM), *Caligula cachara* (CC), *Loepa katinka* (LK) and BM (Figures S2 and S9, SI). Moreover, all degummed non-mulberry silk samples show a characteristic peak at 963 cm^−1^, corresponding to the β-sheet conformation of polyalanine (A)_n_. This peak is specific to wild silk spun, e.g., by Saturniidae silk moths and cannot be observed in spectra of BM silk (see Figure S2)^26,46^. Our results suggest that degummed silk of *Actias selene* (AcS) has the lowest amount of (A)_n_, followed by ArM silk, while a much higher (A)_n_ amount was determined for *Epiphora bauhiniae* (EB), *Callosamia promethea* (CaP), HC and *Samia* silk (Fig. 1). Main characteristic features of degummed BM silk, whose β-sheets consist of polyalanineglycine (AG)_n_ segments, are the peaks at 975 and 998 cm^−1^. As described above (see Degumming of BM cocoons), these bands are clearly visible in the ATR-FTIR spectra of degummed BM silk. Furthermore, it is known from previous studies that the silk of *B. mandarina* has a high amount of (AG)_n_ β-sheets^26^. In contrast, most Saturniidae silks are poor in (AG)_n_ β-sheets. Thus, these characteristic peaks are not observed in the IR spectra of degummed and untreated non-mulberry silk samples. To obtain a further quantitative understanding of the composition of different secondary structures, including β-sheet, β-turn, α-helix and random coil, peak deconvolution analyses were carried out on the amide I bands (1600–1700 cm^−1^, see Refs.^29–31,47^). The deconvolution results revealing the quantitative content of the secondary structure of the degummed silk samples are summarized in Table S6 and Figure S12, SI. The β-sheet content in *Actias*, ArM, AM, CC, LK and *Hyalophora gloveri* (HG) silk is slightly lower compared with APe, AtA, CaP, EB, *Samia* and BM silk. These results show that silk from (semi-)domesticated silkworms such as APe, SCy ricini and BM, in particular, has a higher β-sheet content, which is in good agreement with previous studies^29^. However, an increased β-sheet content can also be found in some wild silks such as EB or CaP. In summary, the analysis of ATR-FTIR spectra enables to discriminate easily between silk of the *Bombyx* genus and Saturniidae silks. Furthermore, some general tendencies can be observed for Saturniidae cocoons and silk, such as a relatively high calcium oxalate content in *Antheraea* cocoons.

#### AA composition of degummed filaments

AA analyses were performed on degummed silks that showed no sericin residues on the filament surface. For this purpose, the silk samples were hydrolyzed with 6 N hydrochloric acid and after derivatization with propyl chloroformate, the AAs were analyzed by GC-FID (see AA analysis by GC-FID and AA analysis by GC-FID—validation experiments). Fifteen AAs were identified and quantified in hydrolysates of degummed silks, namely Ala, Gly, Val, Leu, Ser (Ser + Pro, see validation experiments), Asx (Asp + Asn), Glx (Gln + Glu), Phe, Cys, Lys, His, Tyr, Ile, Met and Thr. It should be noted that the amount of Arg and Trp was not determined and therefore these AAs are not part of the further considerations.

**Figure 2:**
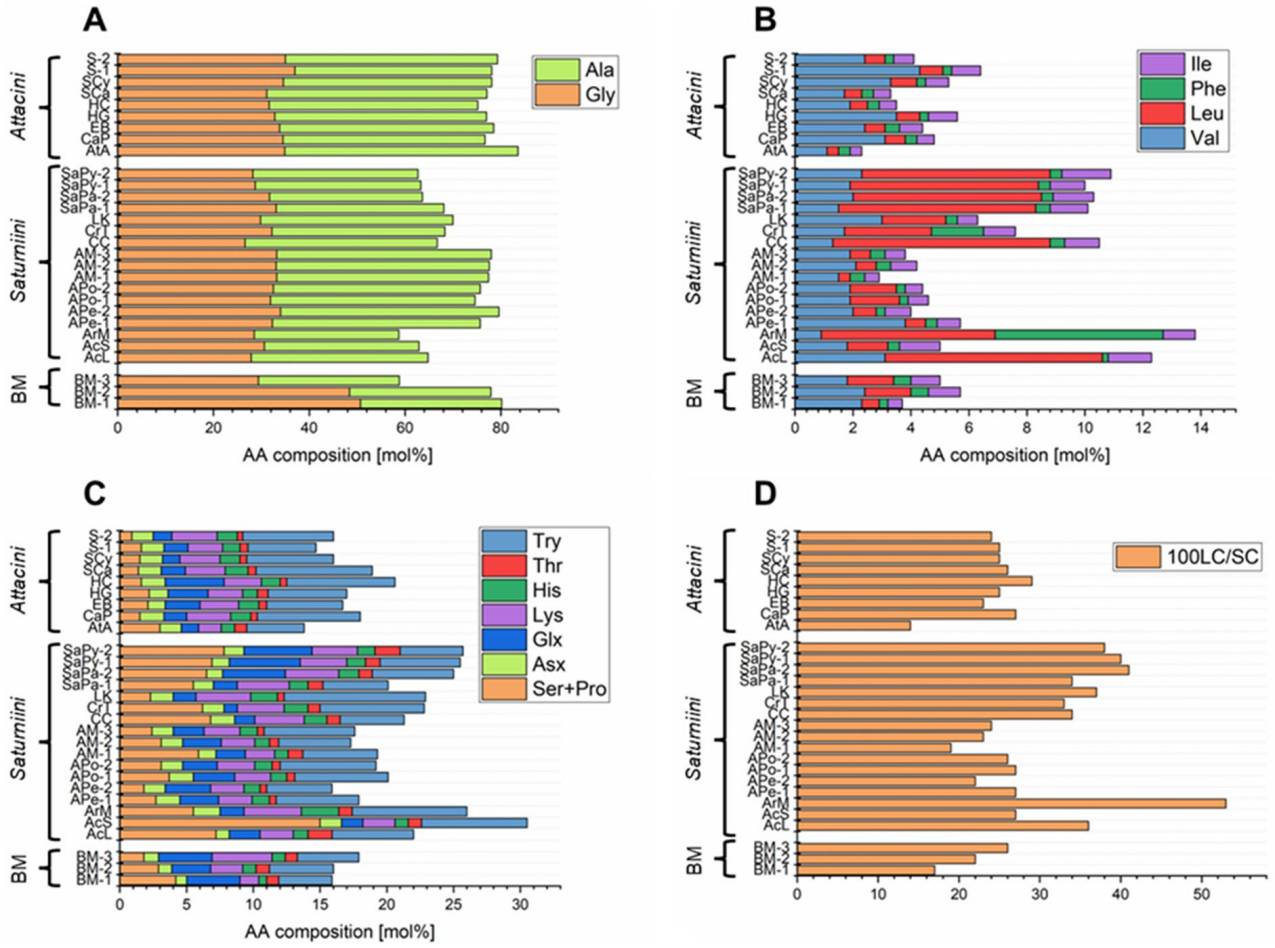
(**A**–**C**) AA composition and the (**D**) 100LC/SC ratio of BM and non-mulberry silks (family Saturniidae), degummed with a mixture of 0.1% Na_2_CO_3_ and 2.5% ethylenediamine at 95 °C (for sample ID assignment see Table 2).

**Figure 3:**
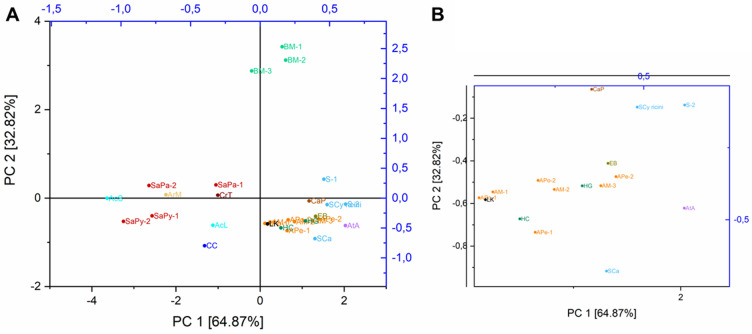
(**A**) PCA score plot of BM and non-mulberry silks (family Saturniidae), degummed with a mixture of 0.1% Na_2_CO_3_ and 2.5% ethylenediamine at 95 °C, (**B**) zoom of the right area (for sample ID assignment see Table 2).

Tables 2 and S4-S5 (SI) and Figs. 2, 3 summarize the AA composition of the studied BM (family Bombycidae) and non-mulberry silks (family Saturniidae), degummed with a mixture of 0.1% Na_2_CO_3_ and 2.5% ethylenediamine. Ala and Gly are predominant in BM and non-mulberry fibroin and account for approximately between 60 and 85 mol% of the total AA content, with Ala content (between 35 and 49 mol%) being higher than Gly content (between 27 and 37 mol%) in non-mulberry silks. Exceptions to this are silk samples of AcS, ArM, *Cricula trifenestrata* (CrT) and *Saturnia pavonia* (SaPa). In these samples, the Ala and Gly content is about 30–35 mol% and the Gly/Ala ratio is thus around 1. In contrast, the Ala content of BM silk is about 30 mol%, while the Gly content is approximately 50 mol% (Table S5, SI). Consequently, the ratio of Gly/Ala for fibroin of non-mulberry species belonging to the family Saturniidae is lower than that for BM, suggesting a difference in the primary structure and/or organization of residues in the fibroin (non-mulberry silk: Gly/Ala ≤ 1; BM: Gly/Ala ≈ 1.6–1.7; Gly/Ala of BM silk in other studies: 1.45^24^). In addition, bulky and/or polar AAs such as Tyr or Leu are more abundant in many of the non-mulberry silks examined than in BM fibroin. For instance, the content of bulky AAs is significantly increased in *Actias luna* (AcL, Tyr: 6.1 mol%, Leu: 7.5 mol%, Phe: < LOQ), ArM (Tyr: 8.6 mol%, Leu: 6.0 mol%, Phe: 5.8 mol%) or *Saturnia pyri* (SaPy, Tyr: 4.7–6.0 mol%, Leu: 6.5 mol%, Phe: < LOQ) fibroin compared to BM fibroin (Tyr: 4.0–4.8 mol%, Leu: 0.6–1.6 mol%, Phe: mostly < LOQ). As reported by various authors these compositional differences have implications for structure that can be described by the long chain/short chain (100LC/SC) ratio^23,27^. Since a high proportion of bulky side groups is found in the amorphous region of silk, this structural index is often used in providing insight on the relative degree of crystallinity in fibroin. The 100LC/SC ratio of BM fibroin is 17–26 which is lower than that of non-mulberry silks such as AcL (100LC/SC: 36), SaPy (100LC/SC: 38–40) or ArM (100LC/SC: 53). A Gly/Ala ratio < 1 and a 100LC/SC ratio higher than for BM silk fibroin has already been described in several studies for wild silks such as silk of *Gonometa* species (Lasiocampidae), suggesting a lower structural regularity and thus a lower rigidity and a higher extensibility compared to BM silk^27^. However, we have found that the 100LC/SC ratio is also in the range 20–25 for some of the non-mulberry silks studied, e.g., EB (100LC/SC: 23) or *Antheraea* species (100LC/SC: 19–27).

**Table 2:** AA composition of non-mulberry (Saturniini and Attacini) as well as BM silks degummed with Na_2_CO_3_ (1 g L^−1^) and ethylenediamine (2.5%) at 95 °C.

Tribe	Genus	Species	Description (Supplier)	Sample ID	Comparative AA composition							
					Ala + Gly [mol%]	Gly/Ala	100LC/SC^a)^	P [mol%]^b)^	NP [mol%]^c)^	P/N^d)^	Ser + Pro + Thr + Tyr [mol%]^e)^	B/A^f)^
*Saturniini*	*Actias*	*Actias luna*	America	AcL	64.8	0.8	36	16	84	0.2	15.0	1.1
		*Actias selene*	Thailand	AcS	62.9	0.9	27	23	77	0.3	23.9	1.1
	*Argema*	*Argema mimosae*	Kenya	ArM	58.7	0.9	53	18	82	0.2	15.1	1.9
	*Antheraea*	*Antheraea pernyi*	China	APe-1	75.7	0.7	27	12	88	0.1	9.4	0.8
			North Korea	APe-2	79.6	0.7	22	11	89	0.1	7.2	0.7
		*Antheraea polyphemus*	North USA	APo-1	74.6	0.7	27	14	86	0.2	11.2	0.8
			North America	APo-2	75.7	0.8	26	13	88	0.1	10.8	1.0
		*Antheraea mylitta*	–	AM-1	77.4	0.8	19	14.1	85.9	0.2	12.6	0.9
			–	AM-2	77.6	0.7	23	12.4	87.6	0.1	9.2	0.8
			Tussar silk moth	AM-3	78.0	0.7	24	11.3	88.7	0.1	9.6	1.0
	*Caligula*	*Caligula cachara*	India	CC	66.7	0.7	34	17	83	0.2	12.6	1.7
	*Cricula*	*Cricula trifenestrata*	India	CrT	68.3	0.9	33	16	84	0.2	14.9	2.1
	*Loepa*	*Loepa katinka*	India	LK	70.0	0.7	37	13	87	0.1	13.3	1.8
	*Saturnia*	*Saturnia pavonia*	Europe	SaPa-1	68.1	0.9	34	16	84	0.2	11.6	1.6
			England	SaPa-2	63.7	1.0	41	20	80	0.2	13.5	0.9
		*Saturnia pyri*	France	SaPy-1	63.4	0.8	40	20	80	0.3	14.0	0.8
			Europe	SaPy-2	62.7	0.8	38	22	78	0.3	14.4	0.7
*Attacini*	*Attacus*	*Attacus atlas*	Jonsdorf (butterfly house)	AtA	83.6	0.7	14	9.9	90.2	0.1	8.2	0.9
	*Callosamia*	*Callosamia promethea*	North America	CaP	76.7	0.8	27	11	89	0.1	9.6	1.4
	*Epiphora*	*Epiphora bauhiniae*	Kenya	EB	78.5	0.8	23	11	89	0.1	8.4	1.1
	*Hyalophora*	*Hyalophora gloveri*	America	HG	77.0	0.7	25	12	89	0.1	8.8	0.8
		*Hyalophora cecropia*	America	HC	75.2	0.7	29	13	87	0.1	10.3	0.7
	*Samia*	*Samia canningii* (wild eri silk moth)	India	SCa	77.0	0.7	26	11	89	0.1	10.7	1.4
		Samia cynthia ricini	Thailand	SCy ricini	78.0	0.8	25	10	90	0.1	8.5	1.5
		Eri silk moth	–	S-1	78.1	0.9	25	10	90	0.1	7.3	1.1
			India	S-2	79.3	0.8	24	10	90	0.1	8.1	1.3
	*Bombyx*	*Bombyx mori*	Chinese web store	BM-1	80.2	1.7	17	12.2	87.9	0.1	9.1	0.4
			Halle (Saale)	BM-2	77.9	1.6	22	11.6	88.4	0.1	8.7	0.9
			China	BM-3	76.5	1.6	26	13.9	86.1	0.2	7.3	1.1

^a^Ratio between long chain (LC: other AAs) and short chain (SC: Ala, Gly, Ser, Thr) AAs; ^b^Polar AAs: acidic, basic and hydroxyl AAs; ^c^Non-polar AAs: other AAs; ^d^Ratio between polar and non-polar AA residues; ^e^AAs with hydroxyl groups: Ser, Thr, Tyr; ^f^Ratio between basic (Lys, His) and acidic (Asx, Glx) AAs.

The AA composition of AcL (Ser + Pro: 7.2 mol%, Thr: 1.8 mol%), ArM (Ser + Pro: 5.5 mol%, Thr: 1.0 mol%) and SaPy (Ser + Pro: 6.9–7.8 mol%, Thr: 1.1–1.9 mol%) silk is also characterized by a high Ser + Pro and Thr content, with the total amount of Ser + Pro, Thr and Tyr (AAs with hydroxyl groups: Ser, Thr, Tyr) ranging from 14 to 15 mol%. Furthermore, a relatively high content of the basic AAs Lys (4.3 mol%) and His (2.8 mol%) was found in the silk samples of ArM. In comparison, the total amount of Lys and His (2.0–5.5 mol%, method 4–2, see Table 1) and AAs with hydroxyl groups (7.3–9.1 mol%, method 4–2, see Table 1) in BM silk is low. The ratio of polar:non-polar (P:NP) AAs in AcL, ArM and SaPy silk samples is approximately 20:80. In contrast, a P:NP ratio of about 10:90 was determined for BM silk as well as for other non-mulberry silks (e.g. silk of *Samia* species). The higher ratio of basic to acidic (B/A) AAs for wild silks compared to BM silk, mentioned at the beginning (see Refs.^23,24^), cannot be confirmed for all silks examined. However, a comparison with the literature values is difficult here, since many wild silks have a high Arg content, which cannot be determined with the chosen method (see Hušek^48^).

In agreement with Lucas et al., it can be stated that there are no precise relationships between the AA compositions of the different fibroins and their biological classifications. However, some generally valid conclusions can be drawn^49^. Within a genus, for example, there is usually a close similarity between the structural indices, which can be useful parameters for inferring physical structure and chemical reactivity of silks. However, several studies have reported comparable structural indices in silk fibroin from different strains. For instance, Lucas et al. describes a high similarity between the AA composition of BM and *Bena prasinana* fibroin, the latter being a small British moth, which belongs to the Cymbidae family^49^. Consequently, for characterization of silks, it is recommended to combine the results of AA analyses with e.g. microscopic and spectroscopic data.

#### Principal component analysis (PCA)

The use of PCA of AA composition contribute to distinguishing between BM and non-mulberry silks. As shown in Fig. 3, *Antheraea* (sample ID: APe-1, APe-2, APo-1, APo-2, AM-1, AM-2, AM-3; marked orange) and *Hyalophora* (sample ID: HG, HC; marked dark green) silks were positioned close to each other, showing that the silks of these species have greater similarity to each other, in relation to silk from the genus *Saturnia* (sample ID: SaPa-1, SaPa-2, SaPy-1, SaPy-2; marked red), *Actias* (sample ID: AcL, AcS; marked light blue) or *Bombyx* (sample ID: BM-1, BM-2, BM-3; marked green). However, with the data set available, it is not possible to distinguish between the tribes *Saturniini* and *Attacini*. Increasing the size of the data set is the subject of future studies.

### AA analysis by GC-FID—validation experiments

The results of the validation study for AA analyses using GC-FID are summarized in Table S1, SI. Retention times of derivatized AAs were determined using AA standard solutions. Chromatograms of hydrolyzed silk samples of non-mulberry and mulberry silkworms as well as blank samples were free from interference. However, coelution is observed for Pro and Ser derivatives. Since the Ser content in BM silk is known to be significantly higher than the Pro content (0.6%^50^), only the Ser content is given for hydrolysates of BM silk. Many non-mulberry silks also contain significantly less Pro than Ser^24,25^. However, for non-mulberry silk, it is reported as Ser + Pro content. Calibration plots (concentration of AA versus peak area ratio of AA and internal standards) are linear in the respective selected range (min 0.6 mM, max 24.8 mM). For all AAs considered, an R^2^ value > 0,973 was obtained, and both LODs and LOQs were considered adequate for the purposes of the present study. The repeatability, expressed by relative standard deviations (RSD), ranged from 0.03% for Phe to 2.5% for His. To determine the method precision, hydrolysis of 10 mg BM silk, degummed by method 4–2 (see Table 1), was processed six times and RSDs of AA concentrations were calculated. The RSD values for the AAs Ala and Gly, which are predominant in fibroin, are satisfactory at 2.2 and 2.1%, respectively. RSD values of low abundant AAs, meaning AAs present in fibroin at < 5 mol%, range from 3.9% for Asx (Asp + Asn) to 9.9% for Val. The Cys and Met concentrations of the hydrolysates are below the LOQ and LOD, respectively. Thus, no values for method precision are given for these AAs. Trp, a low abundant AA of BM silk^50^, is decomposed during treatment with hydrochloric acid (see Rutherfurd and Gilani^51^) and cannot be determined using this method. In addition, it should be noted that Asn and Gln are deaminated during acid hydrolysis to Asp and Glu^51^. Thus, after hydrolysis with 6 N hydrochloric acid, values for Asp and Glu are reported as the sum of the acid and amide derivatives (Asx or Glx). Recovery tests were made by spiking degummed BM silk with defined concentrations of AAs. The recovery rate of the different AAs after hydrolysis and derivatization ranged from 69% for Asx to 120% for Gly with an average recovery rate of 89% at the low, 95% at the medium and 87% at the high concentration level. The stability of a solution with derivatized AAs was evaluated at room temperature over a 24 h period. The tests show that the deviation for the AA concentrations is not higher than 10% after 10 h; within 24 h a maximum deviation of 25% is observed (Figure S13, SI). Consequently, samples were measured within 10 h after derivatization (for storage stability of not derivatized AAs in 0.1 M hydrochloric acid, see Ref^38,51^).

## Conclusion

Degummed silk samples of twenty silkworm species of the family Saturniidae were analyzed for their AA composition. For comparison, cocoons of BM, the most important silk producer for centuries, were degummed and analyzed. In this context, alkaline, acidic, enzymatic and detergent-based degumming methods were tested and degumming ratios determined. A mixture of 0.1% Na_2_CO_3_ and 2.5% ethylenediamine was found to be an effective agent for degumming Saturniidae and BM cocoons. Degummed silks were hydrolyzed with 6 N hydrochloric acid, and after derivatization with propyl chloroformate, AAs were identified and quantified by GC-FID. To evaluate the potential of this method, parameter such as LOD, LOQ, linearity, recovery rate, method precision, and stability of derivatized AAs were determined. The validated method showed a satisfactory overall analytical performance. As expected, the AA composition and resulting structural indices of Saturniidae fibroin filaments differ significantly from those of BM silk. Moreover, major differences were found between some Saturniidae species. Within a genus, however, a high degree of similarity can usually be observed between the structural indices. In addition, the structural indices, such as the long chain/short chain ratio (100LC/SC), which provide information about the relative degree of crystallinity of fibroin, allow conclusions to be drawn about physical and chemical properties of silks. A PCA of AA composition, conducted on this rather small data panel, reveals, for instance, significant differences between *Antheraea*, *Saturnia* and *Bombyx* silk. Chemometric analyses on a larger data set are the subject of future studies. The silk samples were also characterized by optical microscopy and ATR-FTIR spectroscopy. As already outlined in previous studies, both techniques enable to discriminate between BM and Saturniidae silks. In addition, these methods have been successfully used to monitor the degumming process. ATR-FTIR spectroscopic studies also reveal general tendencies for Saturniidae cocoons and silk, such as a low proportion of polyalanine (A)_n_ β-sheets in degummed AcS silk or a relatively high calcium oxalate content in *Antheraea* cocoons.

### Supplementary Information

Supplementary Information.

## Data Availability

The datasets generated and analyzed during the current study are available from the corresponding author upon request.
