# Supplementary material for: Degumming and characterization of Bombyx mori and non-mulberry silks from Saturniidae silkworms
Source: Sci Rep. 2023 Nov 9;13:19504. doi: 10.1038/s41598-023-46474-5 (PMC10636165; doi:10.1038/s41598-023-46474-5)
Supplement: Supplementary file 1 — Supplementary Information. [file 41598_2023_46474_MOESM1_ESM.pdf]

# Degumming and Characterization of *Bombyx mori* and Non-mulberry Silks from Saturniidae Silkworms

**Theresa Schmidt[1], Nils Puchalla[1], Marcel Schendzielorz[1] & Annemarie E. Kramell[1,*]**

[1] Department of Organic Chemistry, Martin-Luther-University Halle-Wittenberg, Kurt-Mothes-Straße 2, 06120 Halle, Germany

corresponding author: annemarie.kramell@chemie.uni-halle.de

**Abbreviations – species:**

AcL - *Actias luna* L.
AcS - *Actias selene* Hübner
AM - *Antheraea mylitta* Drury
APe - *Antheraea pernyi* Guérin-Méneville
APo - *Antheraea polyphemus* Cramer
ArM - *Argema mimosa* Boisduval
AtA - *Attacus atlas* L.
BM - *Bombyx mori* L.
CC - *Caligula cachara* Moore
CaP - *Callosamia promethea* Drury
CrT - *Cricula trifenestrata* Helfer
EB - *Epiphora bauhiniae* Guerin-Meneville
HC - *Hyalophora cecropia* L.
HG - *Hyalophora gloveri* Strecker
LK - *Loepa katinka* Westwood
SCa - *Samia canningii* Hutton
SCy ricini - *Samia cynthia ricini* Boisduval
SaPa - *Saturnia pavonia* L.
SaPy - *Saturnia pyri* Denis & Schiffermüller

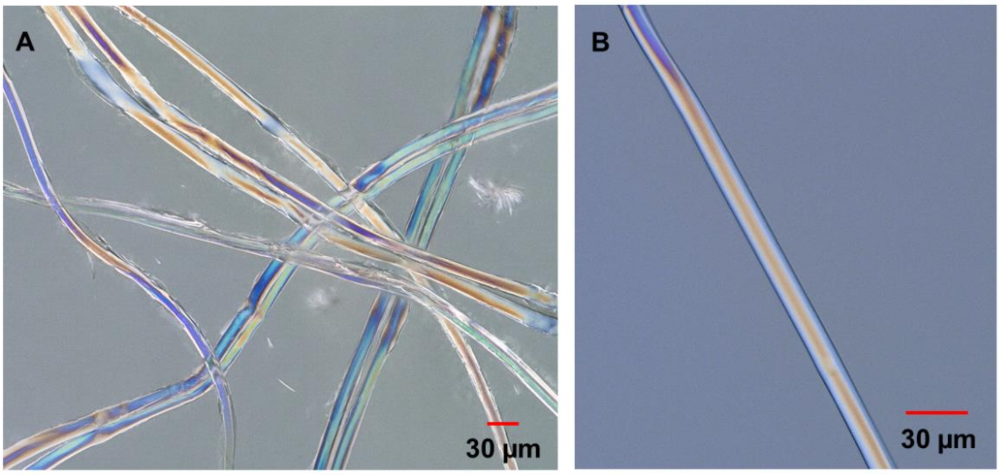


**Fig. S1** Morphologies of BM silk filamtens degummed with (a) 0.1 % citric acid for 120 min at 95 °C (sample ID: BM 5-1; $D_r$ 9 %).; (b) Periquest APG and Perlavin LMO for 60 min at 95 °C (sample ID: BM 7-1; $D_r$ 34 %), see Table S2 for the assignment of the sample ID.

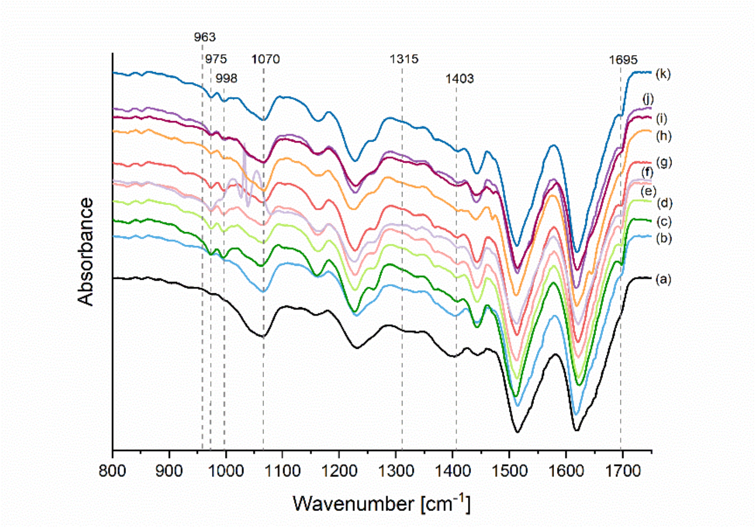


**Fig. S2** ATR-FTIR spectra of untreated BM cocoons: (a) outside surface of cocoons, (b) inside surface of cocoons and BM silk degummed with different degumming agents: (c) BM 4-2, (d) BM 1-2, (e) BM 3-1, (f) BM 7-1, (g) BM 2-2, (h) BM 5-2, (i) BM 6-1, (j) BM 9-2, (k) BM 8-1, see Table S2 for the assignment of the sample ID.

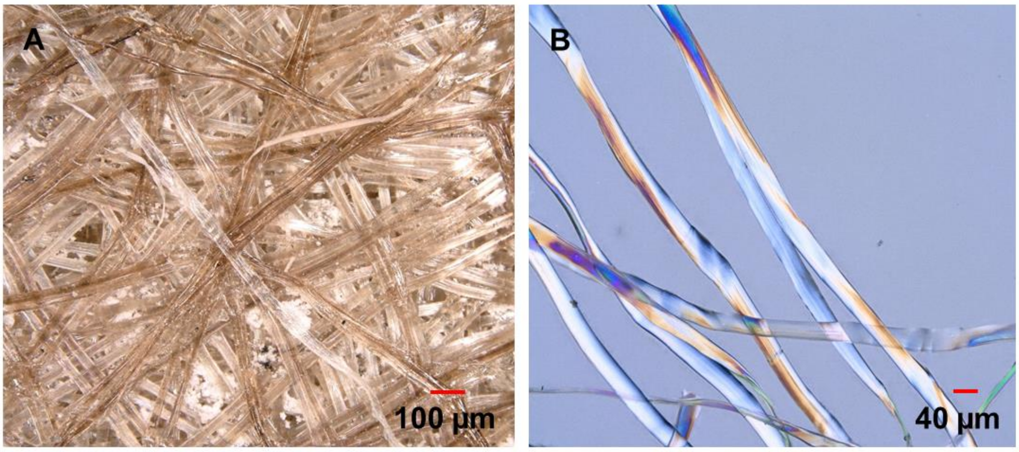


**Fig. S3** Morphologies of AM silk filamtens degummed with (a) Periquest APG and Perlavin LMO for 60 min at 95 °C (sample ID: AM 7-1; $D_r$ 11 %) and (b) a mixture of 2.5 % ethylenediamine and 0.1 % $Na_2CO_3$ for 90 min at 95 °C (sample ID: AM 4-3; $D_r$ 24 %), see Table S3 for the assignment of the sample ID.

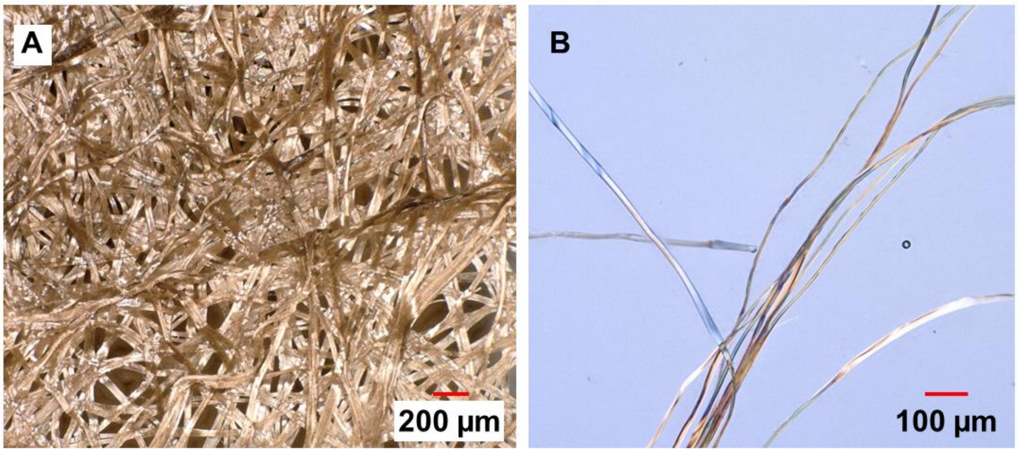


**Fig. S4** Morphologies of AtA silk filamtens degummed with (a) Periquest APG and Perlavin LMO for 60 min at 95 °C (sample ID: AtA 7-2; $D_r$ 18 %) and (b) a mixture of 2.5 % ethylenediamine and 0.1 % $Na_2CO_3$ for 120 min at 95 °C (sample ID: AtA 4-4; $D_r$ 22 %), see Table S4 for the assignment of the sample ID.

**Table S1** Retention time, LOD, LOQ, repeatability (peak area reproducibility at two concentration levels), method precision expressed by RSD % and recovery rates (at three concentration levels) of AAs examined (n.s. … not separated; n.d. … not determined).

| **AA** | **Retention time [min]** | **LOD [pmol]** | **LOQ [pmol]** | **Repeatability RSD [%]** | | **Method precision RSD [%]** | **Recovery [%]** | | |
|---|---|---|---|---|---|---|---|---|---|
| | | | | **concentration level** | | **10 mg BM fibroin** | **concentration level** | | |
| | | | | **low** | **high** | | **low** | **medium** | **high** |
| Ala | 3.60 | 170 | 703 | 0.9 | 0.6 | 2.2 | 92 | 97 | 94 |
| Gly | 3.81 | 88 | 397 | 0.6 | 0.6 | 2.1 | 99 | 74 | 120 |
| Val | 4.15 | 83 | 357 | 0.4 | 0.1 | 9.9 | 74 | 113 | 92 |
| Leu | 4.53 | 19 | 90 | 0.1 | 0.2 | 5.9 | 107 | 121 | 85 |
| Ile | 4.61 | 47 | 195 | 0.3 | 0.7 | 6.8 | 97 | 102 | 75 |
| Thr | 5.02 | 93 | 332 | 0.4 | 0.1 | 4.2 | 79 | 89 | 82 |
| Ser | 5.11 | 69 | 318 | 0.3 | 1.0 | 8.4 | 108 | 114 | 75 |
| Pro | 5.14 | 80 | 304 | 0.1 | 0.1 | n.s. | - | - | - |
| Asp | 6.19 | 50 | 207 | 0.2 | 0.2 | 3.9[a)] | 73 | 69 | 74 |
| Met | 6.28 | 33 | 141 | 0.2 | 0.2 | < LOD | 89 | 103 | 108 |
| Glu | 6.74 | 65 | 286 | 0.4 | 0.5 | 4.5[a)] | 100 | 73 | 72 |
| Phe | 6.86 | 40 | 176 | 0.1 | 0.03 | 7.5 | 76 | 70 | 71 |
| Cys | 7.43 | 88 | 315 | 0.6 | 2.2 | < LOQ | 82 | 97 | 81 |
| Lys | 8.82 | 131 | 513 | 0.5 | 0.6 | 7.4 | 72 | 80 | 78 |
| His | 9.15 | 31 | 140 | 1.0 | 2.5 | 7.3 | 88 | 107 | 78 |
| Tyr | 9.59 | 47 | 198 | 0.8 | 2.1 | 6.7 | 105 | 115 | 116 |
| Trp | 10.13 | 47 | 94 | 0.5 | 0.6 | < LOD | - | - | - |
| Arg | n.d. (see [1,2])[b)] | | | | | | | | |

[a)] Acid hydrolysis converts Asn to Asp and Gln to Glu; [b)] As already described by Hušek, alkoxycarbonyl alkyl esters of Arg are not amenable to GC analysis

**Table S2** AA composition of commercially purchased sericin from BM (family Bombycidae) and BM silks degummed with different degumming agents, expressed as the percentage of the total AA amount.

| Degumming method | Alkaline | | | | | | | | | | | | | Acidic | | | Detergent-based | | | | | Enzyme-based | | | | Silk[b)] | Sericin |
|---|---|---|---|---|---|---|---|---|---|---|---|---|---|---|---|---|---|---|---|---|---|---|---|---|---|---|---|
| | $Na_2CO_3$ | | | $NaHCO_3$ | | Ethylenediamine | | | | | $Na_2CO_3$ and ethylenediamine | | | Citric acid | | | Marseille soap | | Periquest APG and Perlavin LMO | | | Papain | | Pineapple juice | | | |
| Concentration degumming agent | 1 g $L^{-1}$ | | | 0.5 % | 1 % | 2.5 % | | | | 10 % | 1 g $L^{-1}$ ($Na_2CO_3$), 2.5 % (ethylenediamine) | | | 1 g $L^{-1}$ | | 2 g $L^{-1}$ | 1 g $L^{-1}$ | 2 g $L^{-1}$ | 1 g $L^{-1}$ (Periquest APG), 5 g $L^{-1}$ (Perlavin LMO) | | | 1 g $L^{-1}$ | | - | | | |
| Treatment time [min] | 30 | 60 | 90 | 120 | 120 | 30 | 60 | 90 | 120 | 120 | 30 | 60 | 90 | 120 | 240 | 120 | 120 | 120 | 60 | 90 | 120 | 120 | 240 | 120 | 240 | | |
| Sample ID | BM 1-1 | BM 1-2 | BM 1-3 | BM 2-1 | BM 2-2 | BM 3-1 | BM 3-2 | BM 3-3 | BM 3-4 | BM 3-5 | BM 4-1 | BM 4-2 | BM 4-3 | BM 5-1 | BM 5-2 | BM 5-3 | BM 6-1 | BM 6-2 | BM 7-1 | BM 7-2 | BM 7-3 | BM 8-1 | BM 8-2 | BM 9-1 | BM 9-2 | | |
| AA composition [mol%] | | | | | | | | | | | | | | | | | | | | | | | | | | | |
| Ala | 29.3 | 29.4 | 27.7 | 32.8 | 32.3 | 28.3 | 28.3 | 28.4 | 28.2 | 30.3 | 29.5 | 29.5 | 29.7 | 26.6 | 25.7 | 26.8 | 26.0 | 26.6 | 28.6 | 29.0 | 27.7 | 27.4 | 29.6 | 26.9 | 26.1 | 28.0 | 6.5 |
| Gly | 49.0 | 49.4 | 48.6 | 49.4 | 47.3 | 49.7 | 50.7 | 50.3 | 48.6 | 46.4 | 51.7 | 50.7 | 52.1 | 47.3 | 44.8 | 45.5 | 42.9 | 46.0 | 49.6 | 49.0 | 49.1 | 43.4 | 48.1 | 44.9 | 45.6 | 49.2 | 14.3 |
| Val | 2.5 | 2.7 | 2.5 | 2.2 | 1.6 | 2.8 | 2.1 | 2.0 | 2.2 | 3.0 | 2.5 | 2.3 | 2.9 | 3.0 | 2.2 | 3.4 | 2.8 | 3.0 | 2.4 | 2.4 | 2.7 | 3.1 | 3.9 | 3.0 | 3.4 | 2.2 | 2.6 |
| Leu | 0.8 | 0.7 | 0.8 | 0.9 | 0.8 | 0.7 | 0.7 | 0.7 | 0.7 | 1.0 | 0.6 | 0.6 | 0.6 | 1.0 | 1.3 | 1.3 | 1.4 | 1.0 | 0.7 | 0.8 | 0.8 | 1.2 | 1.1 | 1.2 | 1.3 | 0.7 | 1.6 |
| Ser | 4.9 | 4.4 | 6.1 | 3.1 | 3.1 | 4.9 | 4.7 | 4.7 | 5.3 | 5.8 | 4.1 | 4.2 | 4.0 | 9.8 | 10.5 | 9.6 | 9.8 | 7.7 | 5.2 | 5.4 | 5.5 | 9.6 | 6.0 | 11.1 | 10.2 | 5.5 | 35.8 |
| Asx (Asp+Asn) | 0.7 | 0.6 | 0.9 | 1.3 | 1.6 | 0.8 | 0.8 | 0.8 | 0.9 | 0.8 | 0.7 | 0.8 | 0.5 | 0.8 | 0.9 | 0.9 | 1.0 | 1.0 | 0.8 | 0.8 | 0.9 | 0.9 | 0.5 | 0.8 | 0.8 | 0.9 | 2.0 |
| Glx (Glu+Gln) | 4.5 | 4.9 | 4.5 | 2.4 | 3.9 | 4.5 | 4.5 | 4.5 | 4.9 | 4.7 | 3.8 | 4.0 | 3.3 | 4.3 | 5.2 | 5.0 | 6.2 | 5.3 | 4.2 | 4.6 | 4.6 | 5.2 | 4.9 | 4.3 | 4.9 | 5.0 | 4.3 |
| Phe | 0.3[a)] | 0.2[a)] | 0.4[a)] | <LOQ | 0.1[a)] | 0.2[a)] | 0.3[a)] | 0.4 | 0.4 | 0.3[a)] | 0.2[a)] | 0.3[a)] | 0.2[a)] | 0.3[a)] | 0.3[a)] | 0.4[a)] | 0.5 | 0.6 | 0.3[a)] | 0.3[a)] | 0.3[a)] | 0.3[a)] | 0.1[a)] | 0.2[a)] | 0.2[a)] | 0.3[a)] | 0.5[a)] |
| Cys | 0.3[a)] | 0.3[a)] | 0.3[a)] | 0.3[a)] | 0.4[a)] | 0.3[a)] | 0.3[a)] | 0.3[a)] | 0.3[a)] | 0.3[a)] | 0.2[a)] | 0.3[a)] | 0.2[a)] | 0.3[a)] | 0.4[a)] | 0.3[a)] | 0.4[a)] | 0.4[a)] | 0.3[a)] | 0.3[a)] | 0.3[a)] | 0.3[a)] | 0.2[a)] | 0.3[a)] | 0.3[a)] | 0.3[a)] | 1.2[a)] |
| Lys | 1.5 | 1.8 | 1.6 | 1.5 | 1.7 | 1.6 | 1.5 | 1.5 | 1.6 | 1.0 | 1.3 | 1.4 | 1.4 | 1.1 | 1.4 | 0.9 | 1.4 | 1.3 | 1.4 | 1.4 | 1.6 | 1.1 | 0.7 | 1.6 | 0.8 | 1.4 | 6.9 |
| His | 0.7 | 0.7 | 0.7 | 0.6 | 0.7 | 0.7 | 0.7 | 0.7 | 0.8 | 0.4 | 0.6 | 0.6 | 0.6 | 0.4 | 0.5 | 0.4 | 0.6 | 0.5 | 0.6 | 0.7 | 0.7 | 0.4 | 0.3 | 0.4 | 0.4 | 0.7 | 3.5 |
| Tyr | 3.9 | 3.2 | 4.5 | 2.9 | 3.9 | 3.6 | 4.3 | 4.6 | 4.9 | 3.2 | 3.2 | 4.0 | 2.6 | 2.5 | 2.8 | 2.8 | 4.2 | 3.9 | 4.3 | 3.7 | 4.2 | 3.3 | 1.1 | 2.3 | 2.0 | 4.4 | 11.2 |
| Ile | 0.7 | 1.1 | 0.6 | 1.3 | 1.0 | 0.8 | 0.5 | 0.5 | 0.5 | 1.4 | 0.6 | 0.5 | 0.9 | 0.9 | 1.5 | 1.3 | 1.2 | 1.4 | 0.6 | 0.7 | 0.6 | 1.5 | 2.2 | 1.2 | 1.5 | 0.6 | 1.1 |
| Met | < LOD | | | | | | | | | | | | | | | | | | | | | | | | | | 2.1 |
| Thr | 0.9 | 0.7 | 1.0 | 0.8 | 0.9 | 1.1 | 0.7 | 0.8 | 0.8 | 1.3 | 1.0 | 0.9 | 1.0 | 1.6 | 2.6 | 1.5 | 1.6 | 1.5 | 0.9 | 1.0 | 1.0 | 2.3 | 1.3 | 1.9 | 2.5 | 0.8 | 5.7 |
| Trp | < LOD | | | | | | | | | | | | | | | | | | | | | | | | | | |

[a)] Above LOD but just below the LOQ; [b)] Commercially purchased degummed silk filaments of BM (without indication of the degumming method)

**Table S3** AA composition of AM (family Saturniidae, tribe *Saturniini*) silks degummed with different degumming agents, expressed as the percentage of the total AA amount.

| **Degumming method** | **Alkaline** | | | | | **Detergent-based** | |
|---|---|---|---|---|---|---|---|
| | **Ethylenediamine** | | **$Na_2CO_3$ and ethylenediamine** | | | **Periquest APG and Perlavin LMO** | |
| Concentration degumming agent | 2.5 % | | 1 g $L^{-1}$ ($Na_2CO_3$), 2.5 % (ethylenediamine) | | | 1 g $L^{-1}$ (Periquest APG), 5 g $L^{-1}$ (Perlavin LMO) | |
| Treatment time [min] | 60 | 90 | 30 | 60 | 90 | 60 | 90 |
| Sample ID | AM 3-2 | AM 3-3 | AM 4-1 | AM 4-2 | AM 4-3 | AM 7-1 | AM 7-2 |
| **AA composition [mol%]** | | | | | | | |
| Ala | 43.0 | 42.7 | 43.8 | 42.4 | 44.2 | 40.9 | 42.1 |
| Gly | 33.3 | 34.6 | 34.0 | 34.0 | 33.2 | 31.7 | 33.2 |
| Val | 1.4 | 1.6 | 1.4 | 1.5 | 1.5 | 1.9 | 1.6 |
| Leu | 0.5 | 0.5 | 0.5 | 0.5 | 0.4 | 0.8 | 0.6 |
| Ser+Pro | 6.4 | 6.1 | 5.4 | 6.0 | 5.9 | 5.7 | 5.5 |
| Asx (Asp+Asn) | 1.4 | 1.3 | 1.5 | 1.5 | 1.3 | 1.5 | 1.4 |
| Glx (Glu+Gln) | 2.3 | 2.2 | 2.3 | 2.3 | 2.2 | 2.6 | 2.1 |
| Phe | 0.5 | 0.3[a)] | 0.4[a)] | 0.5 | 0.5 | 0.6 | 0.5 |
| Cys | 0.4[a)] | 0.4[a)] | 0.4[a)] | 0.4[a)] | 0.4[a)] | 0.4[a)] | 0.4[a)] |
| Lys | 2.2 | 2.2 | 2.2 | 2.3 | 2.2 | 3.5 | 3.1 |
| His | 1.0 | 0.9 | 1.1 | 1.1 | 1.0 | 1.3 | 1.1 |
| Tyr | 6.5 | 5.3 | 5.9 | 6.2 | 5.6 | 7.5 | 7.0 |
| Ile | 0.4[a)] | 0.6 | 0.5[a)] | 0.5 | 0.5[a)] | 0.6 | 0.5 |
| Met | < LOD | < LOD | 0.1[a)] | < LOD | < LOD | 0.4[a)] | 0.4[a)] |
| Thr | 0.8 | 1.2 | 0.6[a)] | 1.0 | 1.1 | 0.7 | 0.6[a)] |
| Trp | < LOD | | | | | | |

[a)] Above LOD but just below the LOQ

**Table S4** AA composition of AtA (family Saturniidae, tribe *Attacini*) silks degummed with different degumming agents, expressed as the percentage of the total AA amount.

| **Degumming method** | **Alkaline** | | | | | **Detergent-based** | |
|---|---|---|---|---|---|---|---|
| | **Ethylenediamine** | | **$Na_2CO_3$ and ethylenediamine** | | | **Periquest APG and Perlavin LMO** | |
| Concentration degumming agent | 2.5 % | | 1 g $L^{-1}$ ($Na_2CO_3$), 2.5 % (ethylenediamine) | | | 1 g $L^{-1}$ (Periquest APG), 5 g $L^{-1}$ (Perlavin LMO) | |
| Treatment time [min] | 60 | 90 | 60 | 90 | 120 | 60 | 90 |
| Sample ID | AtA 3-2 | AtA 3-3 | AtA 4-2 | AtA 4-3 | AtA 4-4 | AtA 7-1 | AtA 7-2 |
| $D_r$ [%] | 19 | 19 | 16 | 17 | 22 | 16 | 18 |
| **AA composition [mol%]** | | | | | | | |
| Ala | 39.9 | 48.7 | 47.3 | 47.2 | 48.7 | 47.2 | 49.2 |
| Gly | 33.6 | 33.2 | 33.3 | 32.0 | 34.9 | 31.4 | 31.9 |
| Val | 1.4 | 1.2 | 1.2 | 1.2 | 1.1 | 2.5 | 2.8 |
| Leu | 0.3 | 0.4 | 0.4 | 0.2 | 0.4 | 0.9 | 0.8 |
| Ser+Pro | 6.4 | 3.7 | 3.8 | 4.0 | 3.0 | 3.1 | 2.8 |
| Asx (Asp+Asn) | 2.5 | 1.7 | 1.7 | 1.8 | 1.6 | 1.6 | 1.4 |
| Glx (Glu+Gln) | 2.2 | 1.4 | 1.6 | 1.7 | 1.3 | 2.0 | 1.4 |
| Phe | 0.5[a)] | 0.3[a)] | 0.3[a)] | 0.5[a)] | 0.4[a)] | 0.3[a)] | 0.3[a)] |
| Cys | 0.6[a)] | 0.4[a)] | 0.4[a)] | 0.4[a)] | 0.4[a)] | 0.5[a)] | 0.5[a)] |
| Lys | 2.8 | 1.8 | 1.9 | 1.6 | 1.7 | 2.4 | 1.9 |
| His | 1.4 | 1.0 | 1.2 | 1.2 | 1.0 | 1.2 | 1.0 |
| Tyr | 6.1 | 4.8 | 5.2 | 5.8 | 4.3 | 4.6 | 4.0 |
| Ile | 0.6[a)] | 0.7 | 0.5[a)] | 0.7 | 0.4[a)] | 1.0 | 0.9 |
| Met | < LOD | < LOD | < LOD | < LOD | < LOD | 0.5 | 0.5[a)] |
| Thr | 1.7 | 0.8 | 1.2 | 1.5 | 0.9 | 0.9 | 0.8 |
| Trp | < LOD | | | | | | |

[a)] Above LOD but just below the LOQ

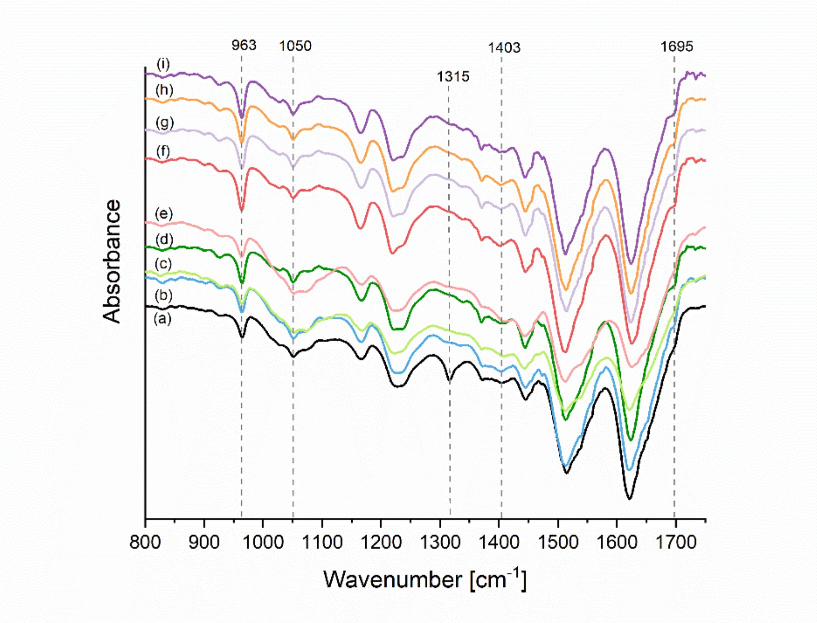


**Fig. S5** ATR-FTIR spectra of untreated AtA cocoons: (a) outside surface of cocoon, (b) inside surface of cocoon and AtA silk degummed with different degumming agents: (c) AtA 7-1, (d) AtA 7-2, (e) AtA 4-4, (f) AtA 4-2, (g) AtA 4-3, (h) AtA 3-2, (i) AtA 3-3, see Table S4 for the assignment of the sample ID.

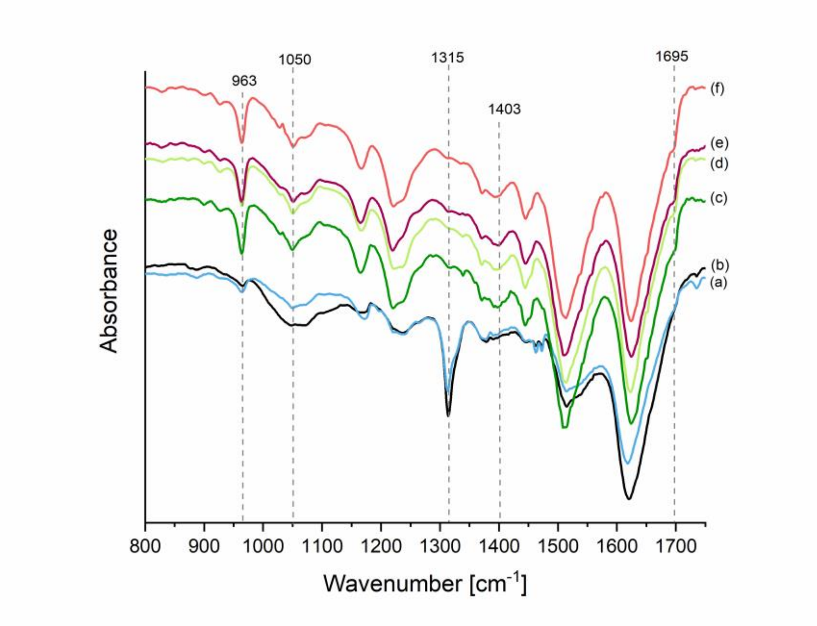


**Fig. S6** ATR-FTIR spectra of untreated AM cocoons: (a) inside surface of cocoon, (b) outside surface of cocoon and AM silk degummed with different degumming agents: (c) AM 4-2, (d) AM 4-3, (e) AM 4-1, (f) AM 7-1, see Table S3 for the assignment of the sample ID.

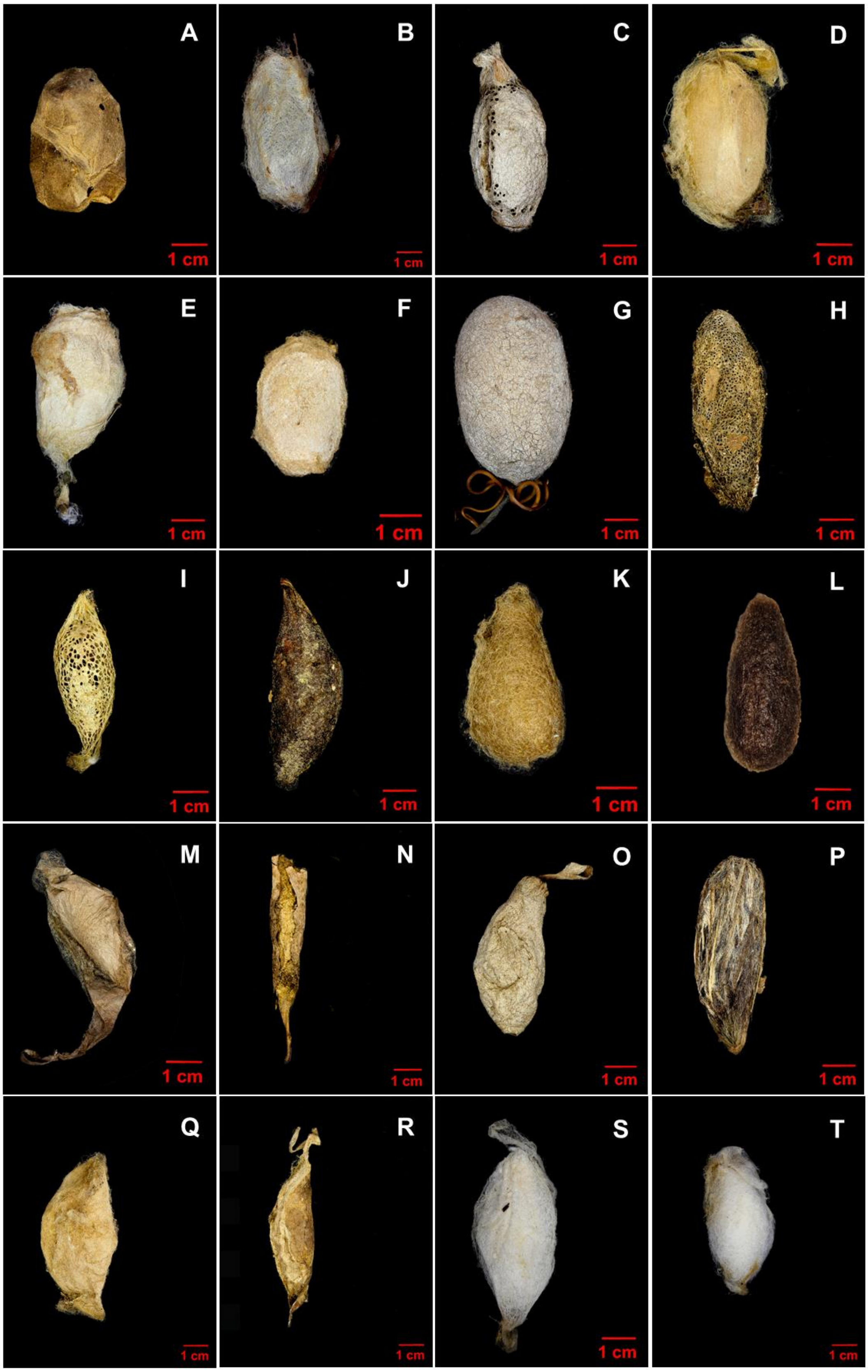


**Fig. S7** (a) AcL, (b), AcS, (c) ArM, (d) APe from China, (e) APe from North Korea, (f) APo, (g) AM, (h) CC, (i) CrT; (j) LK, (k) SaPa and (l) SaPy cocoons from *Saturniini* tribe; (m) AtA, (n) CaP, (o) EB, (p) HG, (q) HC, (r) SCa, (s) Scy ricini and (t) eri silk moth cocoons from *Attacini* tribe.

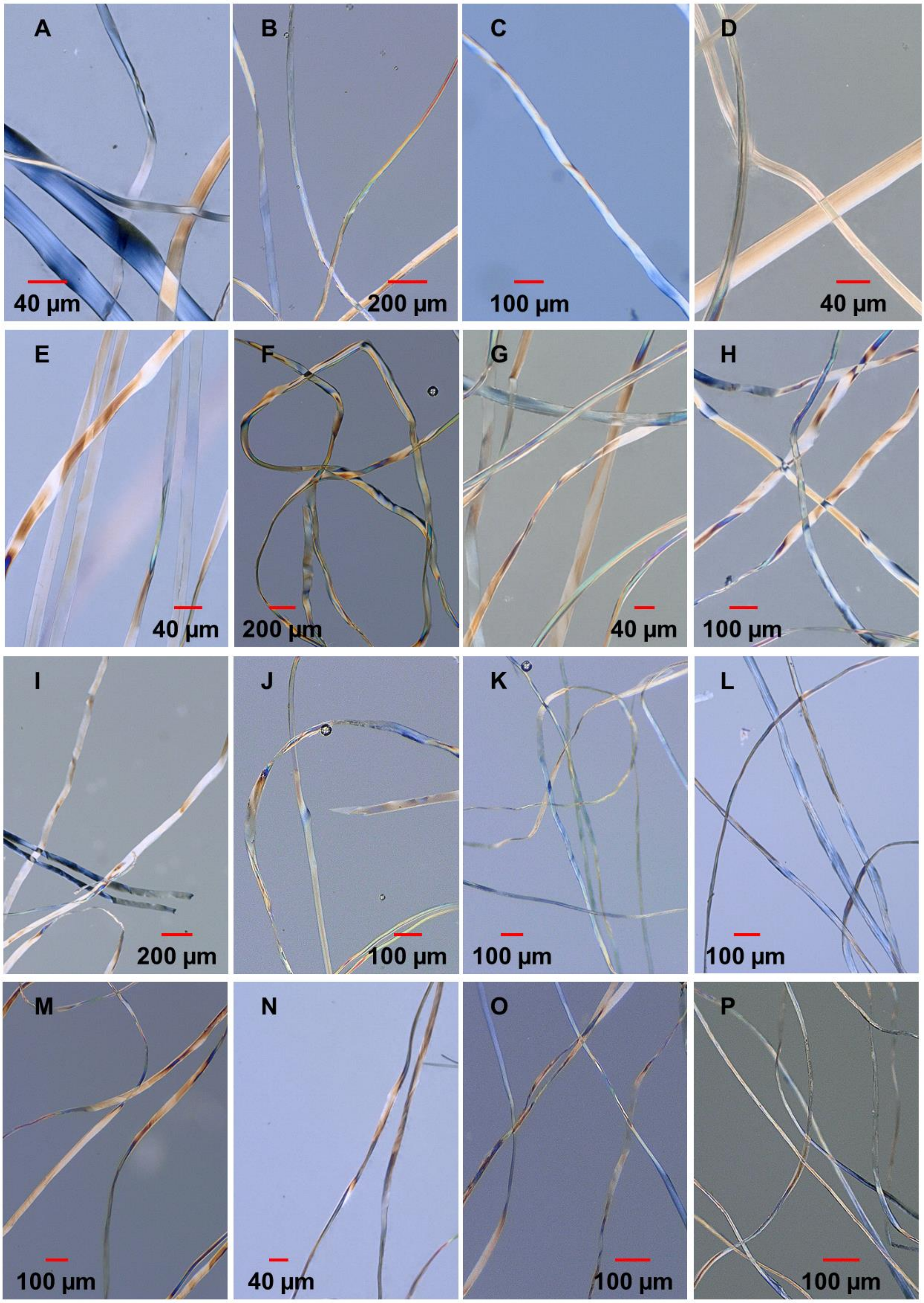


**Fig. S8** Morphologies of silk filamtens degummed with a mixture of 2.5 % ethylenediamine and 0.1 % $Na_2CO_3$ at 95 °C: (a) AcL, (b) AcS, (c) ArM, (d) APe, (e) APo, (f) CC, (g) CrT, (h) LK, (i) SaPa and (j) SaPy from *Saturniini* tribe; (k) CaP, (l) EB, (m) HG, (n) HC, (o) SCa and (p) SCy ricini from *Attacini* tribe.

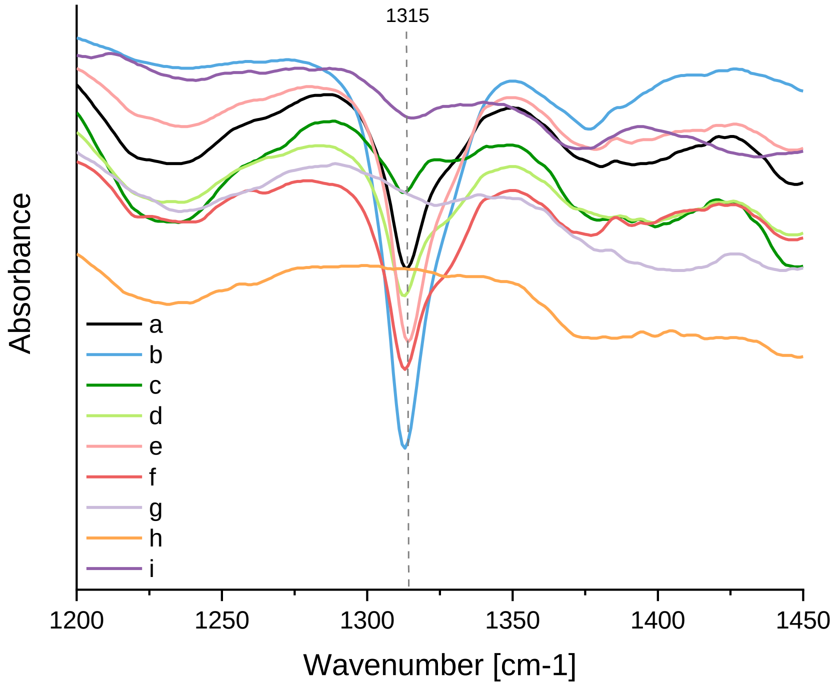


**Fig. S9** ATR-FTIR spectra of untreated cocoons in the range 1200-1450 $cm^{-1}$: (a) APo - inside surface, (b) APo - outside surface, (c) APe - inside surface, (d) APe - outside surface, (e) AM - outside surface, (f) AM - inside surface, (g) ArM - outside surface, (h) CC - outside surface, (i) LK - outside surface.

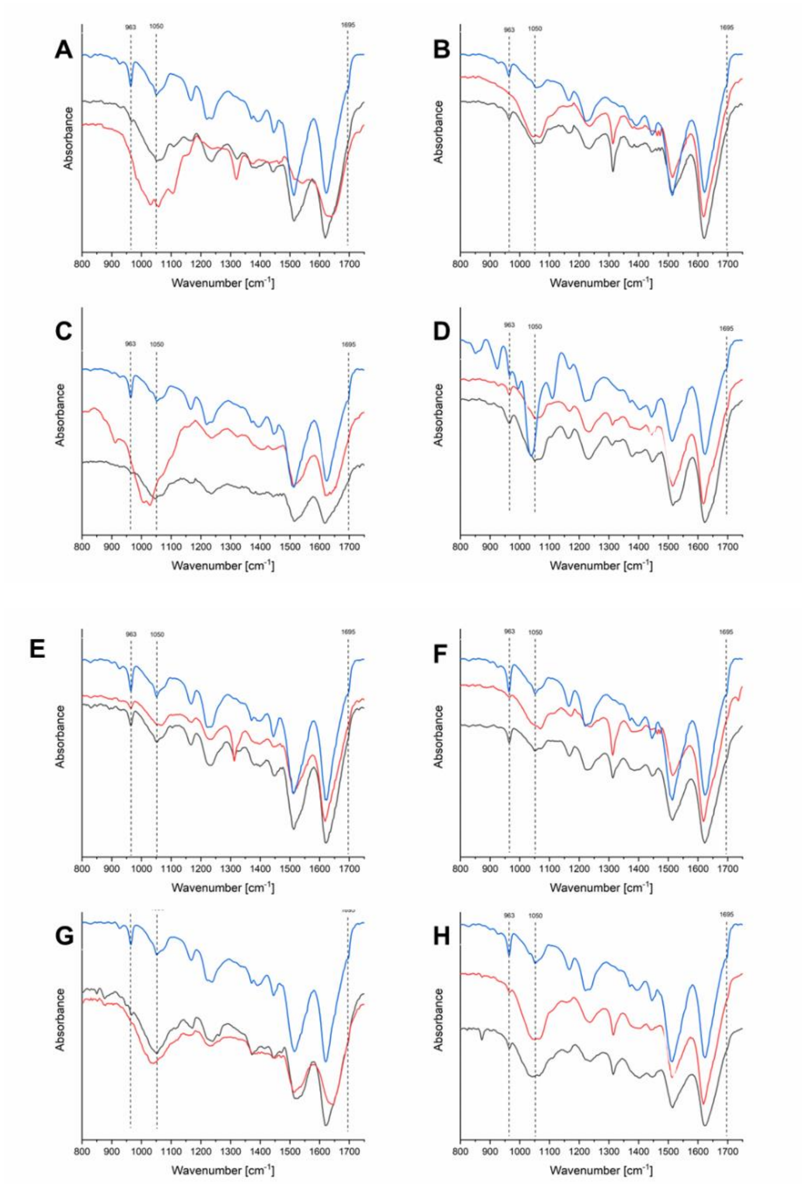


**Fig. S10** ATR-FTIR spectra of untreated cocoons (black graph - inside surface, red graph - outside surface) and degummed silks (blue graph) in the range 800-1750 $cm^{-1}$: (a) AcL, (b) AcS, (c) ArM, (d) APe from China, (e) APe from North Korea, (f) APo, (g) CC and (h) CrT, *Saturniini* tribe.

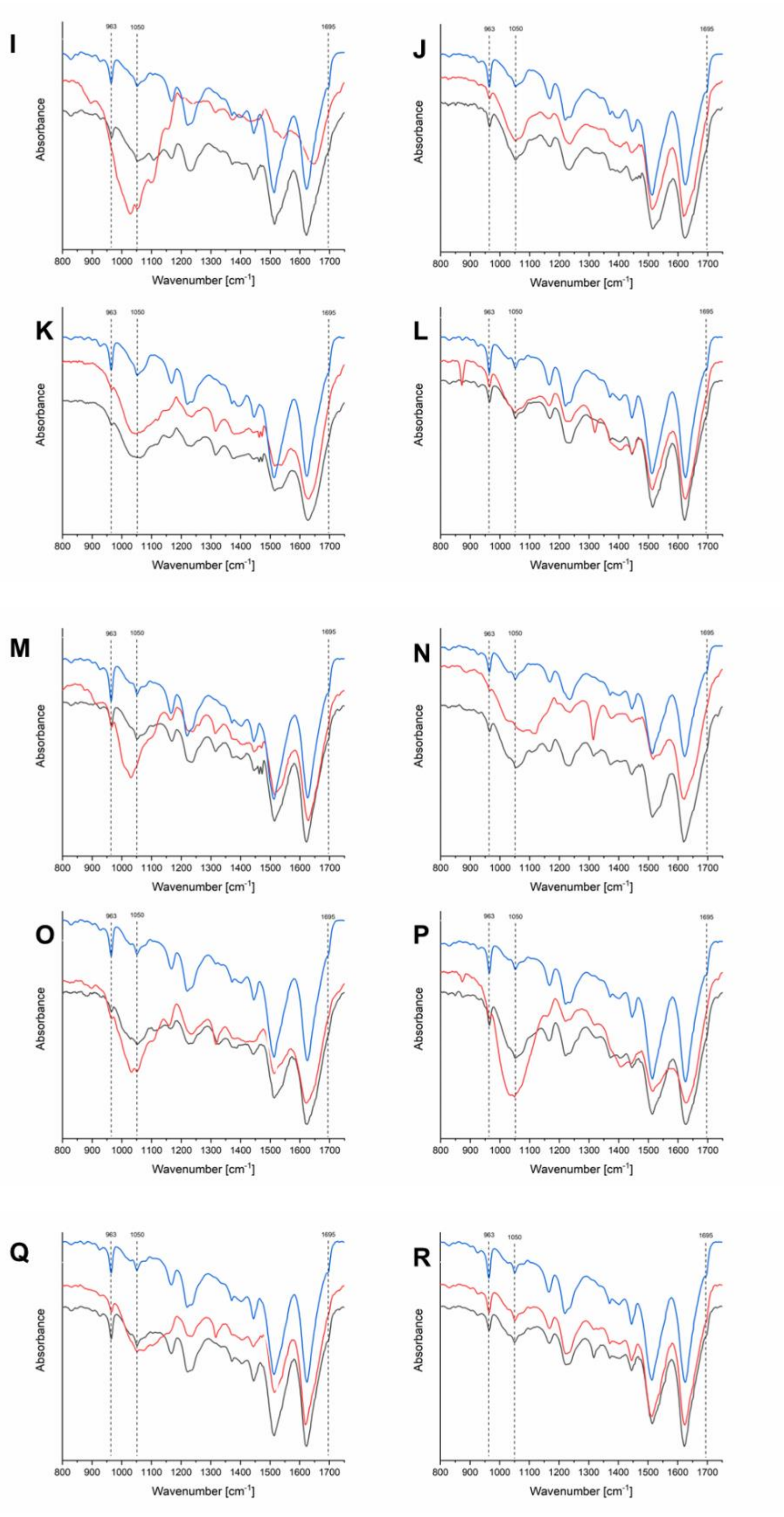


**Fig. S11** ATR-FTIR spectra of untreated cocoons (black graph - inside surface, red graph - outside surface) and degummed silks (blue graph) in the range 800-1750 $cm^{-1}$: (i) LK, (j) SaPa and (k) SaPy, *Saturniini* tribe; (l) CaP, (m) EB, (n) HG, (o) HC, (p) SCa, (q) SCy ricini and (r) eri silk moth, *Attacini* tribe.

**Table S5** Degumming ratio and treatment time of non-mulberry silkworm cocoons of the family Saturniidae and BM cocoons treated with $Na_2CO_3$ (1 g $L^{-1}$) and ethylenediamine (2.5 %) at 95 °C as well as AA composition of the degummed silks.

| Tribe | Species | Description (Supplier) | Sample ID | Treatment time [min] | $D_r$ [%] | AA composition [mol%] | | | | | | | | | | | | | | | |
|---|---|---|---|---|---|---|---|---|---|---|---|---|---|---|---|---|---|---|---|---|---|
| | | | | | | Ala | Gly | Val | Leu | Ser+Pro | Asx (Asp+Asn) | Glx (Glu+Gln) | Phe | Cys | Lys | His | Tyr | Ile | Met | Thr | Trp |
| ***Saturniini*** | *Actias luna* | America | AcL | 90 | 44 | 36.9 | 27.9 | 3.1 | 7.5 | 7.2 | 1.0 | 2.3 | 0.2[a)] | 0.5[a)] | 2.5 | 1.1 | 6.1 | 1.5 | 0.5 | 1.8 | <LOD |
| | *Actias selene* | Thailand | AcS | 60 | 64 | 32.3 | 30.6 | 1.8 | 1.4 | 15 | 1.6 | 1.6 | 0.4[a)] | 0.8[a)] | 2.4 | 1 | 7.9 | 1.4 | 0.8 | 1.0 | |
| | *Argema mimosae* | Kenya | ArM | 90 | 19 | 30.2 | 28.5 | 0.9 | 6 | 5.5 | 2.0 | 1.8 | 5.8 | 0.9 | 4.3 | 2.8 | 8.6 | 1.1 | 0.8 | 1.0 | |
| | *Antheraea pernyi* | China | APe-1 | 60 | 29 | 43.4 | 32.3 | 3.8 | 0.7 | 2.7 | 1.8 | 2.9 | 0.4[a)] | 0.5[a)] | 2.5 | 1.3 | 6.2 | 0.8 | 0.3[a)] | 0.5[a)] | |
| | | North Korea | APe-2 | 90 | 21 | 45.6 | 34.0 | 2 | 0.8 | 1.8 | 1.6 | 3.4 | 0.3[a)] | 0.5[a)] | 2.5 | 1.2 | 4.9 | 0.9 | 0.2[a)] | 0.5[a)] | |
| | *Antheraea polyphemus* | North USA | APo-1 | 60 | 39 | 42.7 | 31.9 | 1.9 | 1.7 | 3.7 | 1.8 | 3.1 | 0.3[a)] | 0.6[a)] | 2.7 | 1.2 | 7.0 | 0.7 | 0.3[a)] | 0.6[a)] | |
| | | North America | APo-2 | 60 | 31 | 43.2 | 32.5 | 1.9 | 1.6 | 3.1 | 1.6 | 2.6 | 0.3[a)] | 0.6[a)] | 2.8 | 1.3 | 7.2 | 0.6 | 0.3[a)] | 0.6[a)] | |
| | *Antheraea mylitta* | - | AM-1 | 90 | 24 | 44.2 | 33.2 | 1.5 | 0.4 | 5.9 | 1.3 | 2.2 | 0.5 | 0.4[a)] | 2.2 | 1.0 | 5.6 | 0.5[a)] | <LOD | 1.1 | |
| | | - | AM-2 | 90 | 17 | 44.5 | 33.1 | 2.1 | 0.7 | 3.1 | 1.6 | 2.9 | 0.5 | 0.5[a)] | 2.5 | 1.1 | 5.4 | 0.9 | 0.4[a)] | 0.7[a)] | |
| | | Tussar silk moth | AM-3 | 90 | 19 | 44.8 | 33.2 | 1.9 | 0.7 | 2.4 | 1.6 | 2.3 | 0.5 | 0.5[a)] | 2.7 | 1.3 | 6.8 | 0.7 | 0.3[a)] | 0.5[a)] | |
| | *Caligula cachara* | India | CC | 240 | 28 | 40.1 | 26.6 | 1.3 | 7.5 | 6.8 | 1.8 | 1.5 | 0.5[a)] | 0.8[a)] | 3.7 | 1.7 | 4.8 | 1.2 | 0.7 | 1.0 | |
| | *Cricula trifenestrata* | India | CrT | 60 | 27 | 36.1 | 32.2 | 1.7 | 3 | 6.2 | 1.6 | 1.0 | 1.8 | 0.9 | 3.5 | 1.8 | 7.8 | 1.1 | 0.5 | 0.9 | |
| | *Loepa katinka* | India | LK | 90 | 22 | 40.2 | 29.8 | 3.0 | 2.2 | 2.3 | 1.7 | 1.7 | 0.4[a)] | 0.6[a)] | 4.1 | 2.0 | 10.6 | 0.7 | 0.3[a)] | 0.5[a)] | |
| | *Saturnia pavonia* | Europe | SaPa-1 | 300 | 21 | 35.0 | 33.1 | 1.5 | 6.8 | 5.5 | 1.5 | 1.8 | 0.5 | 1.0 | 3.9 | 1.4 | 4.9 | 1.3 | 0.7 | 1.1 | |
| | | England | SaPa-2 | 210 | 20 | 31.9 | 31.7 | 2.0 | 6.5 | 6.5 | 1.2 | 4.7 | 0.4[a)] | 0.7[a)] | 4.0 | 1.5 | 6.1 | 1.4 | 0.6 | 1.0 | |
| | *Saturnia pyri* | France | SaPy-1 | 270 | 33 | 34.6 | 28.7 | 1.9 | 6.5 | 6.9 | 1.3 | 5.3 | 0.4[a)] | 0.7 | 3.5 | 1.4 | 6.0 | 1.2 | 0.5 | 1.1 | |
| | | Europe | SaPy-2 | 240 | 28 | 34.5 | 28.2 | 2.3 | 6.5 | 7.8 | 1.5 | 5.1 | 0.4[a)] | 0.8[a)] | 3.4 | 1.3 | 4.7 | 1.7 | <LOD | 1.9 | |
| ***Attacini*** | *Attacus atlas* | Jonsdorf (butterfly house) | AtA | 120 | 22 | 48.7 | 34.9 | 1.1 | 0.4 | 3.0 | 1.6 | 1.3 | 0.4[a)] | 0.4[a)] | 1.7 | 1.0 | 4.3 | 0.4[a)] | <LOD | 0.9 | |
| | *Callosamia promethea* | North America | CaP | 90 | 26 | 42.2 | 34.5 | 3.1 | 0.7 | 1.5 | 1.8 | 1.7 | 0.4[a)] | 0.6[a)] | 3.3 | 1.5 | 7.7 | 0.6 | 0.2[a)] | 0.5[a)] | |
| | *Epiphora bauhiniae* | Kenya | EB | 120 | 19 | 44.7 | 33.8 | 2.4 | 0.7 | 2.1 | 1.3 | 2.6 | 0.5 | 0.4[a)] | 2.9 | 1.5 | 5.7 | 0.8 | <LOD | 0.6[a)] | |
| | *Hyalophora gloveri* | America | HG | 180 | 24 | 44.2 | 32.8 | 3.5 | 0.8 | 2.2 | 1.4 | 3.0 | 0.3[a)] | 0.5[a)] | 2.6 | 1.1 | 5.9 | 1.0 | <LOD | 0.8 | |
| | *Hyalophora cecropia* | America | HC | 60 | 22 | 43.6 | 31.6 | 1.9 | 0.6 | 1.6 | 1.8 | 4.4 | 0.4[a)] | 0.5[a)] | 2.8 | 1.4 | 8.1 | 0.6 | 0.2 | 0.5[a)] | |
| | *Samia canningii* (wild eri silk moth) | India | SCa | 210 | 22 | 46 | 31.1 | 1.7 | 0.6 | 1.4 | 1.7 | 1.8 | 0.4[a)] | 0.5[a)] | 3.0 | 1.7 | 8.7 | 0.6 | 0.3[a)] | 0.6[a)] | |
| | Samia cynthia ricini | Thailand | SCy ricini | 120 | 26 | 43.5 | 34.6 | 3.3 | 0.9 | 1.5 | 1.7 | 1.3 | 0.3[a)] | 0.6[a)] | 3.0 | 1.5 | 6.5 | 0.8 | 0.2[a)] | 0.5[a)] | |
| | Eri silk moth | - | S-1 | 120 | 16 | 41.1 | 37.0 | 4.3 | 0.8 | 1.6 | 1.7 | 1.8 | 0.3[a)] | 0.5[a)] | 2.6 | 1.3 | 5.1 | 1.0 | 0.3[a)] | 0.6[a)] | |
| | | India | S-2 | 120 | 23 | 44.3 | 35.0 | 2.4 | 0.7 | 0.9 | 1.6 | 1.4 | 0.3[a)] | 0.5[a)] | 3.4 | 1.5 | 6.8 | 0.7 | 0.2[a)] | 0.4[a)] | |
| | *Bombyx mori* | Chinese web store | BM-1 | 60 | 31 | 29.5 | 50.7 | 2.3 | 0.6 | 4.2 | 0.8 | 4.0 | 0.3[a)] | 0.3[a)] | 1.4 | 0.6 | 4.0 | 0.5 | <LOD | 0.9 | |
| | | Halle (Saale) | BM-2 | 60 | 29 | 29.5 | 48.4 | 2.4 | 1.6 | 2.9 | 1.0 | 2.9 | 0.6 | 0.4[a)] | 2.4 | 1.0 | 4.8 | 1.1 | <LOD | 1.0 | |
| | | China | BM-3 | 60 | 29 | 29.4 | 47.1 | 1.8 | 1.6 | 1.8 | 1.1 | 4.0 | 0.6 | 0.4[a)] | 4.5 | 1.0 | 4.6 | 1.0 | <LOD | 0.9 | |

[a)] Above LOD but just below the LOQ

**Table S6** Secondary structural characteristics of degummed silks treated with $Na_2CO_3$ (1 g $L^{-1}$) and ethylenediamine (2.5 %) at 95 °C.

| Tribe | Species | Sample ID | β-sheet [%][a)] | β-turn [%][a)] | α-helix/random coil [%][a)] |
|---|---|---|---|---|---|
| ***Saturniini*** | *Actias luna* | AcL | 56.5 ± 1.1 | 11.3 ± 0.5 | 32.1 ± 0.5 |
| | *Actias selene* | AcS | 57.2 ± 1.3 | 11.3 ± 0.9 | 31.6 ± 0.4 |
| | *Argema mimosae* | ArM | 56.6 ± 2.6 | 11.7 ± 1.2 | 31.6 ± 1.5 |
| | *Antheraea pernyi* | APe-1 | 62.9 ± 2.3 | 9.4 ± 0.1 | 27.6 ± 2.1 |
| | | APe-2 | 64.1 ± 5.6 | 9.3 ± 0.8 | 26.6 ± 4.8 |
| | *Antheraea polyphemus* | APo-2 | 60.2 ± 0.4 | 9.8 ± 0.2 | 30.0 ± 0.4 |
| | *Antheraea mylitta* | AM-1 | 56.7 ± 1.9 | 11.6 ± 0.8 | 31.7 ± 1.1 |
| | | AM-3 | 56.6 ± 2.2 | 11.9 ± 1.4 | 31.5 ± 0.8 |
| | *Caligula cachara* | CC | 57.2 ± 1.1 | 11.3 ± 0.7 | 31.5 ± 0.5 |
| | *Cricula trifenestrata* | CrT | 59.2 ± 2.8 | 10.3 ± 1.0 | 30.5 ± 1.8 |
| | *Loepa katinka* | LK | 56.4 ± 1.2 | 11.4 ± 0.6 | 32.2 ± 0.6 |
| | *Saturnia pavonia* | SaPa-2 | 58.6 ± 0.9 | 10.4 ± 0.4 | 31.0 ± 0.6 |
| | *Saturnia pyri* | SaPy-2 | 56.7 ± 2.2 | 11.4 ± 1.3 | 31.9 ± 1.0 |
| ***Attacini*** | *Attacus atlas* | AtA | 66.9 ± 6.1 | 8.8 ± 0.9 | 24.2 ± 5.2 |
| | *Callosamia promethea* | CaP | 60.3 ± 0.8 | 10.1 ± 0.4 | 29.6 ± 0.3 |
| | *Epiphora bauhiniae* | EB | 62.1 ± 1.3 | 9.5 ± 0.7 | 28.3 ± 0.8 |
| | *Hyalophora gloveri* | HG | 57.6 ± 0.7 | 10.3 ± 0.2 | 32.1 ± 0.6 |
| | *Hyalophora cecropia* | HC | 60.1 ± 2.4 | 9.8 ± 1.0 | 30.1 ± 1.7 |
| | *Samia canningii* (wild eri silk moth) | SCa | 64.6 ± 3.3 | 9.2 ± 0.5 | 26.3 ± 3.6 |
| | Samia cynthia ricini | SCy ricini | 60.9 ±0.8 | 9.5 ± 0.4 | 29.7 ± 0.5 |
| | *Bombyx mori* | BM-1 | 62.3 ± 0.9 | 8.7 ± 0.5 | 28.9 ± 0.5 |
| | | BM-3 | 66.4 ± 2.8 | 6.9 ± 1.5 | a.7 ±1.4 |

[a)] Deconvolution results of amide I band in ATR-FTIR spectra (n = 3-4)

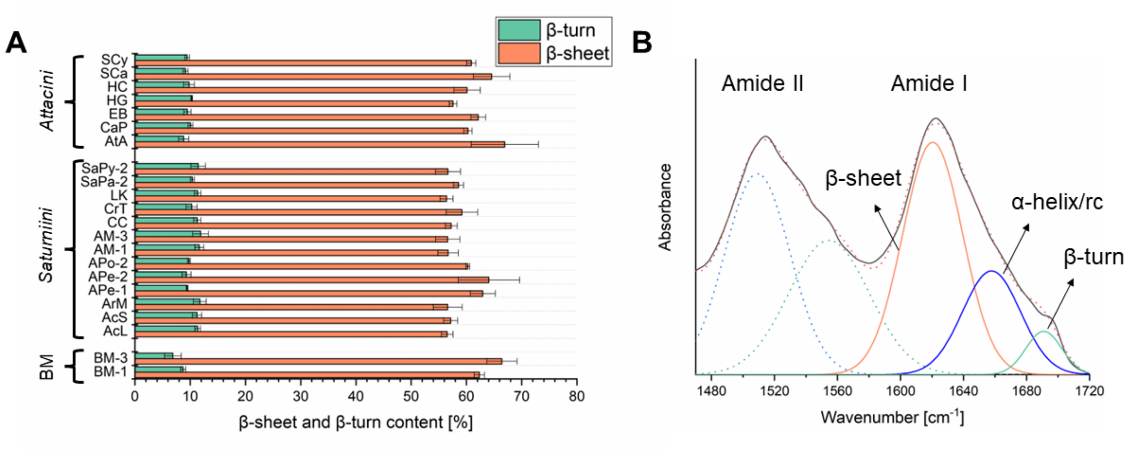


**Fig. S12** (a) β-sheet and β-turn content of degummed silks treated with $Na_2CO_3$ (1 g $L^{-1}$) and ethylenediamine (2.5 %) at 95 °C. (b) Deconvolution result of amide I band of degummed APe silk: deconvoluted peaks with centers at 1620 $cm^{-1}$ (β-sheet conformation), 1658 $cm^{-1}$ (random coil/helical conformation) and 1691 $cm^{-1}$ (β-turn); black solid curve, original spectrum; red dashed curve, simulated spectrum from summed peaks.

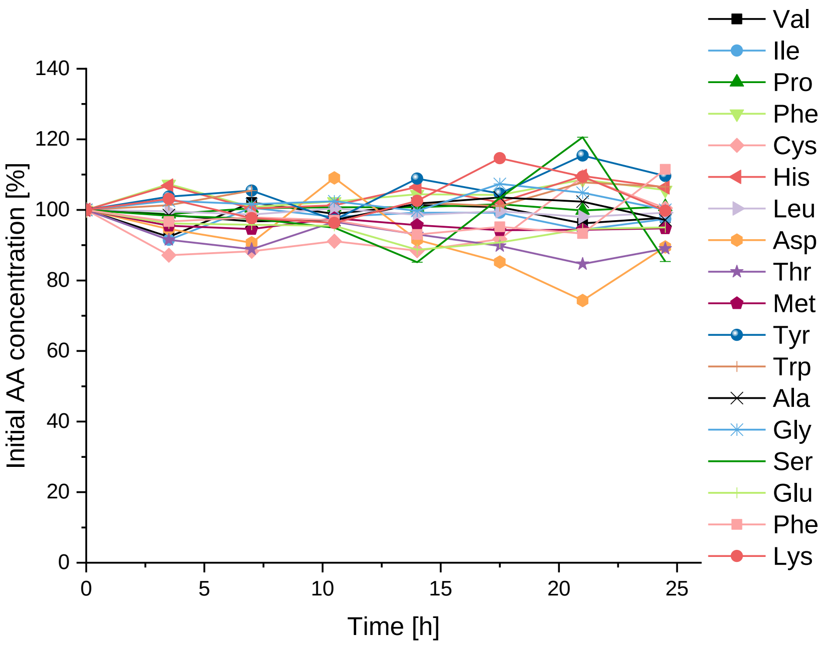


**Fig. S13** Variation of AA derivatives concentration stored at room temperature over a 24 hour period.

## References


1. Hušek, P. Rapid derivatization and gas chromatographic determination of amino acids. *J. Chromatogr. A* **552**, 289-299, doi:https://doi.org/10.1016/S0021-9673(01)95945-X (1991).
2. Hušek, P. Chloroformates in gas chromatography as general purpose derivatizing agents. *J. Chromatogr. B* **717**, 57-91, doi:https://doi.org/10.1016/S0378-4347(98)00136-4 (1998).
